# PGC1α Loss Promotes Lung Cancer Metastasis through Epithelial-Mesenchymal Transition

**DOI:** 10.3390/cancers13081772

**Published:** 2021-04-08

**Authors:** Taek-In Oh, Mingyu Lee, Yoon-Mi Lee, Geon-Hee Kim, Daekee Lee, Jueng Soo You, Sun Ha Kim, Minyoung Choi, Hyonchol Jang, Yeong-Min Park, Hyun-Woo Shin, Dong Hoon Shin, Ji-Hong Lim

**Affiliations:** 1 Department of Biomedical Chemistry, College of Biomedical & Health Science, Konkuk University, Chungju 27478, Korea; dk1050@kku.ac.kr (T.-I.O.); yoonmilee@kku.ac.kr (Y.-M.L.); rlarjsgml4@kku.ac.kr (G.-H.K.); 2 Department of Applied Life Science, Graduate School, BK21 Program, Konkuk University, Chungju 27478, Korea; 3 Obstructive Upper airway Research (OUaR) Laboratory, Department of Pharmacology, Seoul National University College of Medicine, Seoul 03080, Korea; alligator08@snu.ac.kr; 4 Department of Biomedical Sciences, Seoul National University Graduate School, Seoul 03080, Korea; 5 Cancer Research Institute, Seoul National University College of Medicine, Seoul 03080, Korea; 6 Department of Life Science, Ewha Womans University, Seoul 03760, Korea; daekee@ewha.ac.kr; 7 Department of Biochemistry, School of Medicine, Konkuk University, Seoul 05029, Korea; jsyou@kku.ac.kr; 8 Research Institute, National Cancer Center, Department of Cancer Biomedical Science, National Cancer Center Graduate School of Cancer Science and Policy, Goyang 10408, Korea; 1905205@ncc.re.kr (S.H.K.); cmy413@ncc.re.kr (M.C.); hjang@ncc.re.kr (H.J.); 9 Department of Immunology, School of Medicine, Konkuk University, Seoul 05029, Korea; immun3023@kku.ac.kr; 10 Diabetes and Bio-Research Center, Konkuk University, Chungju 27478, Korea

**Keywords:** lung cancer, PGC1α, ID1, TCF4, TWIST1, EMT, metastasis

## Abstract

**Simple Summary:**

Despite the therapeutic advances, lung cancer is the most dangerous cancer with poor 5-year survival rate due to metastasis and recurrence. Accumulated evidence indicates that the epithelial–mesenchymal transition (EMT) is considered to be responsible for the lung cancer metastasis; however, the transcriptional frameworks that regulate EMT-related gene expression are still poorly understood. Here, we suggest that cooperation of TCF4-TWIST1 controlled by the PGC1α-ID1 transcriptional axis mediates EMT and lung cancer metastasis, and that this molecular framework is an attractive target for lung cancer diagnosis and treatment.

**Abstract:**

PGC1α oppositely regulates cancer metastasis in melanoma, breast, and pancreatic cancer; however, little is known about its impact on lung cancer metastasis. Transcriptome and in vivo xenograft analysis show that a decreased PGC1α correlates with the epithelial–mesenchymal transition (EMT) and lung cancer metastasis. The deletion of a single Pgc1α allele in mice promotes bone metastasis of Kras^G12D^-driven lung cancer. Mechanistically, PGC1α predominantly activates ID1 expression, which interferes with TCF4-TWIST1 cooperation during EMT. Bioinformatic and clinical studies have shown that PGC1α and ID1 are downregulated in lung cancer, and correlate with a poor survival rate. Our study indicates that TCF4-TWIST1-mediated EMT, which is regulated by the PGC1α-ID1 transcriptional axis, is a potential diagnostic and therapeutic target for metastatic lung cancer.

## 1. Introduction

Lung cancer is the most common type of cancer to metastasize to the bones. Secondary bone tumors are often observed in approximately 30–40% of lung cancer patients during the course of their disease [1]. Thus, understanding the molecular mechanism by which lung cancer metastasizes to bones will reveal molecular targets for the treatment of bone metastasis in lung cancer and improve the patients’ quality of life (QOL).

The epithelial–mesenchymal transition (EMT) is required for activating invasiveness and metastasis, which is considered a hallmark of cancer in most solid tumors of the epithelial origin: Lung, colorectal, breast, prostate, and pancreatic cancer [2,3]. Multiple types of transcription factors, such as TWIST1, SNAIL, SLUG, ZEB1, and ZEB2, are required for EMT [4]. TWIST1 was originally identified as a basic helix–loop–helix (bHLH) transcription factor that determines cell fate during embryonic development and morphogenesis [5,6]. However, the molecular framework by which TWIST1 and its transcriptional complex regulate EMT gene expression is still not understood. As an endogenous EMT promoting stimulus, tumor growth factor b1 (TGFβ1), which derives from cancer-associated fibroblasts (CAFs), promotes EMT through several signaling pathways, including the TGFβR-SMADs axis [7]; however, this mechanism is still not fully understood.

PPARGC1A (PGC1α) is emerging as a potential target for cancer treatment due to its functional role being closely linked to cancer development [8,9], chemoresistance [10], and distant metastasis [9,11,12]. It has been reported that PGC1α suppresses distant metastasis by mediating a transcriptional circuit comprising the inhibitor of DNA binding 2 (ID2) and transcription factor 4 (TCF4, also called ITF-2 or E2-2), independently of metabolic reprogramming in melanoma [12]. In addition, an increased rate of tumor growth and metastasis has been associated with altered metabolic features observed in Pten and Pgc1α knock-out driven prostate cancer [9]. On the contrary, PGC1α promotes breast cancer metastasis along with an increased mitochondrial oxidative phosphorylation and an altered bioenergetics flexibility [11]. An association has been observed between a deregulated PGC1α expression, such as decreased PGC1α levels in breast, prostate, melanoma, and renal cell carcinoma (RCC), and a poor prognosis [9,12,13,14]. Conversely, a correlation between an increased PGC1α level in proliferative melanoma and breast cancer and a poor outcome has been observed [11,15,16]. Recent evidence revealed that PGC1α is closely associated with lung fibrosis through metabolic energetics and TGFβ1-mediated transcriptional axis [17,18,19,20]. In addition, it has been reported that decreased PGC1α mRNA levels are correlated with poor survival in patients with non-small-cell lung carcinoma (NSCLC) [21]. However, the functional role and mechanisms of PGC1α during lung cancer development and progression are largely unknown. In the present study, we thus investigated the association between PGC1α expression and lung cancer progression.

TCF4 is a transcription factor that belongs to class I bHLH (also called E-protein), which is expressed in different tissues such as brain, muscle, liver, and lung [22], and it is associated with T lymphocyte development [23] and differentiation of neuronal progenitors [24]. Several pieces of evidence have shown the tumor suppressor role of TCF4, in suppressing the adenoma–carcinoma transition, tumorigenesis, and progression in colorectal cancer [25,26,27]. In contrast, the oncogenic role of TCF4 has been shown by inducing cell proliferation in colorectal cancer [28], EMT in MDCK normal kidney cells [22], and distant metastasis in malignant melanoma [12]. Nevertheless, the functional and mechanistic role of TCF4 as an oncogene or a tumor suppressor in lung cancer development and progression has not been investigated.

The ID family in vertebrates (ID1, ID2, ID3, and ID4), which belongs to the helix–loop–helix (HLH) domain containing transcription factors, mainly controls cell fate determination, differentiation, and cell proliferation [29]. Mechanistically, ID proteins, which lack a DNA-binding domain, act as endogenous inhibitors of the protein–protein interaction between E-protein transcription factors, such as TCF3 (E2A), TCF4, and TCF12 (HEB), and bHLH transcription factors [30]. Consistent with the mechanistic action of ID proteins, the ID family has been demonstrated to regulate many biological processes in human cancers, such as the cell cycle, proliferation, chemoresistance, metastasis, and angiogenesis [31]. Indeed, the deregulated IDs mRNA and protein expression levels have been observed in human cancers and are often associated with poor prognosis [30,31,32]. However, the mechanistic frameworks by which IDs regulate lung cancer development and progression, including their downstream effectors, remain poorly understood. This understanding would be critical for the development of a drug target for lung cancer.

In the present study, we investigated the functional role of PGC1α and its regulatory mechanism in lung cancer metastasis via EMT to understand and elucidate the underlying molecular network for potential therapeutic avenues.

## 2. Materials and Methods

### 2.1. RNA-seq, GSEA, and Core Enrichment Gene Set Analysis

RNA was isolated from control or PGC1α knocked-down A549 cells by use of TRIzol (Invitrogen, Carlsbad, CA, USA). RNA-Seq library construction was performed using TruSeq Stranded mRNA LT Sample Prep Kit and sequenced following NovaSeq6000 System User Guide at Macrogen (Seoul, South Korea). Enrichment analysis for RNA-Seq data was carried out using the standard tool of gene set enrichment analysis (GSEA). Enrichment analysis was conducted using the GSEA software v4.03 and a formatted GCT file was used as input for the GSEA algorithm (available from: http://www.broadinstitute.org/gsea (accessed on 1 August 2019). In the analysis, the gene sets of the Hallmarks collection (i.e., 50 gene sets representing well-defined biological processes) and Curated gene set collection from v7.0 molecular signature database (mSigDB C2 category; http://software.broadinstitute.org/gsea/msigdb (accessed on 1 August 2019) were used, and the number of permutations was set to 1000. After GSEA, core enrichment genes (*p* < 0.05 and fold change > 1.5), which contribute to the leading-edge subset, are selected and visualized by using the Multiple Experiment Viewer software (MEV). The gene sets with *p* < 0.05 and false discovery rate (FDR) < 0.25 were considered significantly enriched.

### 2.2. Bioinformatic Analysis Using Human Lung Cancer Biopsies

PPARGC1A (PGC1α) and ID1 mRNA expressions in 26 different types of cancers were obtained from the cBioPortal (www.cbioportal.org (accessed on 1 January 2020)) where mRNA data were offered in the form of normalized RSEM value. The number of patients are as follows: chRCC, kidney chromophobe (*n* = 66); liver, liver hepatocellular carcinoma (*n* = 366); cholangiocarcinoma (*n* = 36); ccRCC, kidney renal clear cell carcinoma (*n* = 469); glioblastoma (*n* = 154); glioma (*n* = 514); GBM, glioblastoma multiforme (*n* = 160); thyroid, thyroid carcinoma (*n* = 498); colorectal, colorectal adenocarcinoma (*n* = 382); melanoma, skin cutaneous melanoma (*n* = 443); pancreas, pancreatic adenocarcinoma (*n* = 177); Thymoma (*n* = 119); esophageal, esophageal cancer (*n* = 181); ACC, adrenocortical carcinoma (*n* = 78); uterine CS, uterine carcinosarcoma (*n* = 57); prostate, prostate adenocarcinoma (*n* = 493); sarcoma (*n* = 253); lung ade, lung adenocarcinoma (*n* = 230); cervical, cervical squamous carcinoma (*n* = 294); mesothelioma (*n* = 87); head and neck, head and neck squamous cell carcinoma (*n* = 279); bladder, bladder urothelial carcinoma (*n* = 129); breast, breast invasive carcinoma (*n* = 1100); testicular germ cell, testicular germ cell cancer (*n* = 156); ovarian, ovarian serous cystadenocarcinoma (*n* = 307); lung Squ, lung squamous cell carcinoma (*n* = 178). Publicly available microarray datasets (GSE85841, GSE7670, GSE102511, GSE19804, GSE19188, GSE14407, GSE66957, GSE26910, GSE108757) were downloaded from Gene Expression Omnibus (www.ncbi.nlm.nih.gov/geo (accessed on 1 January 2020) and analyzed to compare mRNA levels of PGC1α and ID1 between normal and cancer tissues. The 1569141_a_at probe or 219195_at probe (corresponding to PGC1α), and the 208937_s_at probe (corresponding to ID1) were used to determine the value of PGC1α and ID1, respectively. Lung tissues (*n* = 113) were grouped as three classes: Normal lung adjacent to cancers (*n* = 9), primary lung cancers (*n* = 94), and metastatic lung cancers (*n* = 10). For survival analysis, the median expression values of each protein from patient microarray results were used as criteria standard to distinguish the low expression and high expression groups. For correlation analysis, spearman’s correlation test was performed to analyze the relationship between paired genes. The R values indicate the existing linear correlation between two variables X and Y, giving a value between +1 and −1, where 1 is total positive correlation, 0 is no correlation, and −1 is total negative correlation. The *p* value represents the significance of this R coefficient.

### 2.3. Human Lung Cancer Patient Samples and Immunohistochemistry

Human lung cancer tissue arrays were purchased from Super Bio Chips Lab (Seoul, South Korea). Clinical information on lung cancer is delineated in Table S2. The array tissue slides were dried for 2 h in an oven at 60 °C, autoclaved for antigen retrieval, treated with 3% H_2_O_2_, incubated with primary antibodies (against PGC1α, ID1, or E-cadherin) overnight at 4 °C. The slides were biotinylated with a secondary antibody for 1 h at room temperature. The immune complexes were visualized using Polink-2 Plus HRP Broad Kit with DAB (GBI Labs, WA, USA) following the manufacturers protocol, and tissue slides were counterstained with Mayer’s hematoxylin (Sigma-Aldrich, St. Louis, MO, USA) for 10 min. Three randomly selected high-power fields (HPFs) were viewed and photographed. The expression level was determined based on intensity and nuclear positive cell number and scored from 0 to 5.

### 2.4. Tumor Xenograft in Mice and Serial Dilution In Vivo

Single-cell suspensions were prepared in a media:Matrigel (1:1) mixture and subcutaneously injected in 100 μL volumes. Sample size was determined by degree of dilution as follows: Four mice/experimental group for 10^3^ cells, six mice/experimental group for 10^4^ cells, eight mice/experimental group for 10^5^ cells, five mice/experimental group for 10^6^ cells, and three mice/experimental group for 10^7^ cells dilution conditions. Nude mice were injected at a dorsal flank site with A549 lung cancer cells in dilution conditions. Tumor volume was measured with calipers (volume = L × w × w × 0.52, where L is the width at the widest point of the tumor and w is the width perpendicular to L). When tumor reached a volume of 500 mm^3^, mice we were counted as tumor bearing mice.

### 2.5. Lung Orthotopic and Tail Vein Injection Xenograft Model

All animal procedures were performed in accordance with a protocol approved the Institutional Animal Care and Use Committee (IACUC) of National Cancer Center Research Institute. NCCRI is an Association for Assessment and Accreditation of Laboratory Animal Care International (AAALAC International) accredited facility and abide by the Institute of Laboratory Animal Resources (ILAR) guide and Usage Committee (NCC-19-312). Nude mice (BALB/cAnNCrj-nu/nu) from Charles River Japan Inc. (Shin-Yokohama, Japan) were anesthetized with isoflurane via inhalation in an enclosed box chamber. Mice were positioned in a supine position and the jaw and tongue were drawn away from the esophageal region using forceps while inserting a 22-gauge Hamilton TLC syringe (1705, Hamilton, Reno, NV, USA) into the trachea. The glass light is administrated on the mouse’s upper chest and injected with 1 × 10^6^ A549 lung cancer cells suspended in 50 μL of phosphate-buffered saline (PBS). After instillation, the mouse was allowed to recover under visual control before placement back into the cage for a predetermined period after exposure. To do tail vein injection, the mice were warmed by placing in a cage under a heat lamp for 10 min to dilate the veins and then place the mice in the restraining device of appropriate size. Swab the tail with a gauze dampened with alcohol to increase the visibility of the vein. 1 × 10^6^ A549 lung cancer cells in PBS 100 μL were injected intravenously into one of the two lateral tail veins in the middle third of the tail. Non-invasive bioluminescence imaging was performed 20, 40, and 60 days after injection to quantify the primary and metastatic tumor burden using IVIS Lumina XRMS In vivo Imaging System (PerkinElmer, Akron, OH, USA).

### 2.6. Kras Transgenic Mice

Kras transgenic mice (129S/Sv-Kras^tm3Tyj/J^, stock number 008185) were purchased from The Jackson Laboratory (Sacramento, CA, USA). Mice homozygous for Kras^G12D^ allele are lethal. Heterozygote mice develop tumors in the lungs with 100% incidence, which are first detectable as pleural nodules at eight weeks of age.

### 2.7. Generation of Ppargc1α (Pgc1α) knock-Out Mouse Using CRISPR-Cas9

C57BL/6J female mice were superovulated with intraperitoneal (IP) injection of 5 I.U. pregnant mare serum gonadotropin (Merck KGaA, Darmstadt, Germany) followed by human chorionic gonadotropin (Merck KGaA) 48 h later, then bred to C57BL/6J male mice. Next morning, one-cell embryos were obtained from the oviduct and cultured in the microdrop of KSOM media (Merck KGaA) under the mineral oil (Merck KGaA) until microinjection as described elsewhere. Cas9 mRNA was prepared with mMESSAGE mMACHINE™ T7 ULTRA Transcription Kit (ThermoFisher, AM1345) according to the manufacturer’s protocol using pST1374-NLS-flag-linker-Cas9 (Addgene, #44758) as a template DNA. Ppargc1α (Pgc1α) sgRNA was prepared with MEGAshortscript™ T7 Kit (ThermoFisher, AM1354) according to the manufacturer’s protocol. The target sequence for Pgc1α sgRNA is 5′-ATTGTAGCTGAGCTGAGTGTTGG-3′ (underline indicates PAM sequence). Cas9 mRNA (10 ng/μL) and sgRNA (20 ng/μL) dissolved in 10 mM Tris-HCl, pH 7.4, 0.1 mM EDTA were microinjected into the cytoplasm of one-cell embryos and the embryos that survived microinjection were transferred into the oviducts of pseudopregnant ICR females anesthetized with IP injection of 2.5% Avertin (Merck KGaA). Initial screening of Indel mutation was performed with PCR using DNA extract from F0 mice and agarose gel electrophoresis. Only the mutant mice were bred to C57BL/6J mice and the nature of Indel mutation was further characterized with PCR using DNA extract from F1 heterozygotes mice. The PCR product was subcloned into the TA cloning vector as per the manufacturer’s protocol (Promega, Fitchburg, WI, USA) and analyzed by sequencing. The primer sequences for PCR are 5′-TTTCCCTTTTTCTGGTATGTGTC-3′ (sense), 5′-TTTGCTGCATGGTTCTGAGT-3′ (antisense). We bred four F0 mice and obtained the F1 heterozygous mouse with 11-bp deletion. This deletion gave rise to the premature stop codon as shown in Figure S3. The heterozygotes mice with 11-bp deletion (Ppargc1αem1dkl/KorI) were maintained by backcrossing with C57BL/6J mice 2 to 3 more generations before breeding with Kras^G12D^ mice. The PCR genotyping results in 112-bp specific for wild-type allele and 101-bp specific for mutant-type allele, respectively. All mice experiments were approved by the Institutional Animal Care and Use Committee (IACUC) at Ewha Womans University (2015-01-072).

### 2.8. FDG-Position Emission Tomography/Computed Tomography (PET/CT) Scanning

Mice in this study were assigned to four different groups and age-matched. Group 1 is PGC1α^WT^; Kras^WT^. Group 2 is PGC1α^+/−^; Kras^WT^. Group 3 is PGC1α^WT^; Kras^G12D^. Group 4 is PGC1α^+/−^; Kras^G12D^. PET/CT scanning was performed after mice were fasted for 12 h but had free access to water. Mice were anesthetized using vaporized isoflurane (4% for induction; 2.5% for maintenance). Sterile normal saline (0.1 mL) was injected subcutaneously to ensure adequate hydration. PET/CT scanners (Biograph LOS; Siemens Healthcare, Erlangen, Germany and Discovery LS; GE Healthcare, Milwaukee, WI, USA). Non-contrast CT images were acquired in the range of the skull base to upper thigh, and subsequent PET images were acquired 60 min after lateral tail vein injection of 18F-FDG (8.65 ± 2.7 MBq). The standardized uptake value (SUV) was calculated as (decay-corrected activity [kBq] per milliliter of tissue volume)/body mass [g]). The SUVs of lesions were obtained by manually placing of a volume of interested (VOI) around the lesion. Acquisition of dynamic spiral CT imaging was performed using a multidetector CT scanner (Lightspeed pro-16, GE Healthcare) with contrast enhancement.

### 2.9. Cell Culture, Lentiviral Transduction and Generation of Stable Cell Lines

H1437 and H460 lung cancer cells were obtained from the American Type Culture Collection (Manassas, VA, USA) and A549, H1299, Calu-3, Calu-1, H358, H1650, and H1666 lung cancer cells were obtained from the Korean Cell Line Bank (Seoul, Korea). Lung cancer cells were cultured in Dulbecco’s modified Eagle’s medium (DMEM) and Roswell Park Memorial Institute (RPMI) 1640 containing 10% fetal bovine serum (FBS) and antibiotics. STR analysis of A549, H358, and Calu-1 were performed by the Korean Cell Line Bank (Seoul, South Korea). HEK293T cells were cultured in DMEM and used for the production of lentiviral particles, and the examination of ectopically expressed protein-protein interaction. For the generation of Myc-tagged TCF4 overexpressing A549 and H358 cells, the pcDNA-empty or Myc-TCF4 construct was transfected by using Lipofectamine 2000 (Invitrogen, Carlsbad, CA, USA), and transfected cells were selected by G418 (500 μg/mL) for 7 days. The pLX304-V5 vector was used for the generation of V5-tagged GFP, PGC1α, ID1, or TCF4 overexpressing A549 cells. HEK293T cells were transfected by using Polyfect transfection reagent (Qiagen, Hilden, Germany) with an envelope vector (pMD2.G), packaging vector (psPAX2), and pLX304-V5 vectors encoding GFP, PGC1α, ID1, or TCF4. Transfected HEK293T cells were incubated for 48 h with 30% FBS containing DMEM to allow amplification of lentiviruses, and lentiviral particles were then concentrated and purified by using Millipore (Burlington, MA, USA) Lentivirus Purification kit.

### 2.10. Quantitative Real-Time PCR (qRT-PCR) for Measurement of Gene Expression

A quantitative real-time polymerase chain reaction (qRT-PCR) was carried out as described [12]. Briefly, total RNA was isolated with TRIzol (Invitrogen, Carlsbad, CA, USA) and chloroform, and the RNA pellet was washed by 75% ethanol and resolved in DEPC-treated water. Two micrograms of total RNA was used for cDNA synthesis using a hig-capacity cDNA reverse transcription kit (Applied Biosystems, Foster City, CA, USA). Quantitative PCR was performed using SYBR Green PCR Master Mix (Applied Biosystems, Foster City, CA, USA). Relative mRNA expression was calculated versus human 36B4 expression. The sequences of qPCR primers (5′-3′) are listed in Table S3.

### 2.11. Western Blotting and Co-Immunoprecipitation

The detailed procedure for Western blotting was described previously [12]. Briefly, protein samples were prepared by using a cell lysis buffer containing 1% IGEPAL, 150 mM NaCl, 50 mM Tris-HCl (pH 7.9), 10 mM NaF, 0.1 mM EDTA, and protease inhibitor cocktail. For co-immunoprecipitation, HEK293T cells were transiently transfected with the interested protein expression vector using Polyfect (Qiagen, Hilden, Germany), and then transfected cells were incubated for 24 h to allow time for protein expression and molecular networking. Cell lysates were prepared using 1% CHAPS and 150 mM NaCl containing lysis buffer, and then lysates were incubated with anti-Flag, anti-ID1, anti-TCF4, or anti-Myc with protein A/G agarose beads for 24 h at 4 °C. For endogenous protein interactions between TWIST1 and TCF4, 5 mg of the total protein samples were incubated with 10 µg of anti-TWIST1 with protein A/G agarose beads for 24 h at 4 °C. Protein complexes were washed with wash buffer (200 mM NaCl) three times. Eluted proteins were subjected into SDS-PAGE, and then separated proteins were transferred onto a PVDF membrane (Millipore, Burlington, MA, USA). Membranes with separated proteins were incubated with primary antibodies (1:1000~5000) in 5% bovine serum albumin containing 0.05% Tween-20 overnight at 4 °C, and HRP-conjugated secondary antibodies (1: 10,000~20,000) were incubated for 1 h at room temperature. Proteins levels were visualized using an ECL Prime kit (GE healthcare, Milwaukee, USA). Antibodies’ information for Western blotting and co-immunoprecipitation are listed in Table S4.

### 2.12. In Vitro Migration and Invasion Assay

Transwell chambers were purchased from Sigma-Aldrich (St. Louis, MO, USA) and used for in vitro migration and invasion assay. For the in vitro migration assay, control or PGC1α knocked-down A549 (2 × 10^4^) or H358 (2 × 10^4^) cells were seeded with 0.1 mL of FBS-free medium into the upper chamber and further incubated for 12 h. The membrane of the upper side of the Transwell chamber was coated with 8 μM Matrigel (BD Bioscience, San Diego, CA, USA) as an extracellular matrix, and A549 (2 × 10^4^) or H358 (2 × 10^4^) cells were then seeded with 0.1 mL of FBS-free medium into the upper chamber and further incubated for 24 h. The migrated or invaded cells that attached on the lower side of the membrane of the Transwell chamber were then fixed for 10 min with 4% paraformaldehyde and stained for 5 min with hematoxylin and eosin (H&E). The migrated or invaded cells attached on the membrane of the Transwell chamber were placed on a glass slide, and the total cell numbers were quantified from four random fields under 40× magnification with a Nikon Eclipse TS2 (Nikon, Japan).

### 2.13. Transient Transfection

A549 lung cancer cells were seeded at 5 × 10^4^ cells/well in 6-well tissue culture plates and incubated for 24 h to allow attachment and stabilization. Cells were transfected with either 10 nM siRNA targeting human TCF4 mRNA or control (Santa Cruz Biotechnology, Santa Cruz, CA, USA), respectively. For luciferase assay, HEK293T cells were seeded at 10,000 cells/well in 12-well plates, and cells were then transiently transfected with ID1, ID2, E-cadherin-WT, or E-cadherin-Mut-promoter luciferase vector (100 ng). For co-immunoprecipitation assay, HEK293T cells were seeded at 5 × 10^4^ cells/well in 6-well plates, and cells were then transfected with Myc-tagged TCF4, Flag-tagged TWIST1, or ID1-expressing plasmids as following the experimental design. Transient transfection was performed using Polyfect transfection reagent (Qiagen, Hilden, Germany).

### 2.14. Chromatin Immunoprecipitation and Polymerase Chain Reaction (ChIP-PCR)

Chromatin immunoprecipitation (ChIP) assay was performed by using EZ-ChIP assay kit (Millipore, Burlington, MA, USA) according to the manufacturer instructions and slight modifications. Briefly, A549 lung adenocarcinoma cells were incubated for 24 h in the absence or presence of TGFβ1 and expression of V5-GFP or V5-ID1, and then cells were fixed in 1% formaldehyde for 10 min at room temperature. The final concentration of 125 mM glycine was added for quenching formaldehyde for 10 min, and then cells were rinsed twice with cold PBS. Cells were scrapped and collected in PBS containing protease inhibitors and 1 mM phenylmethanesulfonyl fluoride (PMSF). The cell pellets were resuspended in ChIP-lysis buffer containing sodium dodecyl sulfate (SDS), protease inhibitor cocktail, and PMSF, and then chromatins were sheared by sonication with an ultrasonic homogenizer (Bandelin Electronic, Berlin, Germany) for four cycles of 5 min (30 s on, 30 s off upon 30% of power). Samples were diluted in ChIP-dilution buffer containing protease inhibitor cocktail and PMSF, and then samples were incubated with primary antibodies (anti-TCF4, anti-TWIST1, anti-PGC1α, anti-H3K4-me3, and anti-RNA polymerase II) overnight at 4 °C. Immune complexes were recovered with protein A or G agarose beads (Millipore, Burlington, MA, USA) preblocked with salmon sperm DNA (Millipore, Burlington, MA, USA), and then samples were extensively washed by immune complex wash buffer followed by low salt (0.15 M NaCl), high salt (0.5 M NaCl), lithium chloride (0.25 M LiCl), and Tris-EDTA (TE) buffer, respectively. Immunoprecipitated DNA was then isolated with a phenol:chloroform:isoamyl alcohol (25:24:1) as previously described [12]. Immunoprecipitated DNA levels were analyzed by quantitative real-time PCR with specific primers for the promoter region of CDH1, CDH2, and ID1, and DNA enrichments were then normalized by calculation as percent of input. Sequences of PCR primers for the promoter region of CDH1, CDH2, and ID1 were described in Table S3.

### 2.15. Luciferase Assay

Promoter region of ID1 and ID2 were amplified using genomic DNA isolated from A549 lung cancer cells and PCR containing primers with Xho I and Hind III restriction site, and then amplified PCR fragments were inserted into the pGL3-basic vector. Primer sequences for PCR amplification were described in Table S3. E-cadherin-wild type (pXP2-E-cadherin-WT) and E-cadherin-mutant (pXP2-E-cadherin-Mut-E1/E2/E3) luciferase vector were kindly provided by Dr. Muh-Hwa Yang [33]. Each luciferase vector was transfected using a Polyfect (Qiagen, Hilden, Germany) into HEK293 cells with Flag-PGC1α, Flag-TWIST1, Myc-TCF4, or pcDNA-ID1 following the experimental aims, then transfected cells were stabilized for 24 h. Cell lysates were reacted with the luciferase assay buffer, and luciferase activity was measured by using Luminometer (BioTek, Winooski, VT, USA). The transfection efficiency was normalized by β-gal assay.

### 2.16. Cell Viability and Annexin-V Assay

Crystal violet was purchased from Sigma-Aldrich (St. Louis, MO, USA) and used for the cell viability assay [34]. Control or PGC1α knocked-down A549 cells were seeded at 5 × 10^3^ cells/well in 96-well tissue culture plates and incubated for 24 h to allow stabilization. At the following day, cultured cells were incubated with cisplatin, doxorubicin, 5-fluoruracil (5-FU), or paclitaxel at different concentrations, and cells were then further incubated for 3 days. After drug treatment, cells were washed with phosphate-buffered saline (PBS) and fixed with 4% paraformaldehyde, and then cells were stained using crystal violet solution for 20 min at room temperature. Optical density of crystal-violet-stained cells were measured at 570 nm by using an absorbance reader (BioTek, Winooski, VT, USA) (OD570). To measure apoptotic cell death, Annexin-V staining was carried out using Muse™ Annexin V and Dead Cell Assay kit (Millipore, Burlington, MA, USA) [34]. Control or PGC1α knocked-down A549 cells were cultured at 1 × 10^5^ cells/well in 6-well tissue culture plates for 24 h, and cells were then further incubated in the absence or presence of cisplatin (2 μM) for 48 h. Cells were washed using cold PBS pellets and incubated with 100 μL of Muse™ Annexin V and Dead Cell kit reagents (Millipore, Burlington, MA, USA) for 20 min at room temperature. The apoptotic cells population was measured by using Mini Flow Cytometry Muse™ Cell Analyzer (Millipore, Burlington, MA, USA).

### 2.17. Statistical Analysis

All analyses were conducted using SPSS 23.0 software (SPSS, Chicago, IL, USA) and illustrative figures were generated using Prism version 5 (GraphPad Software Inc., San Diego, CA, USA) and SigmaPlot version 10.0 (Systat Software Inc., San Jose, CA, USA). Data are represented as means ± standard errors or mean ± standard deviations. We used non-parametric Mann–Whitney U-test to compare mRNA expression levels and protein expression levels of human specimens. The correlation between target proteins were analyzed using Spearman’s p statistic. Survival analyses in tissue microarray were performed by drawing curves and determining log-rank P test utilizing the Kaplan–Meier method. Data from cell-based experiments including qRT-PCR, ChIP, luciferase assay, and quantitation of invaded and migrated cell numbers are expressed as the mean ± SD, and statistical significance are calculated by Student’s *t*-test, as indicated in the figure legends. *p* < 0.05 was considered statistically significant.

## 3. Results

### 3.1. PGC1α Loss Promotes EMT in Lung Cancer Cells

**Figure 1:**
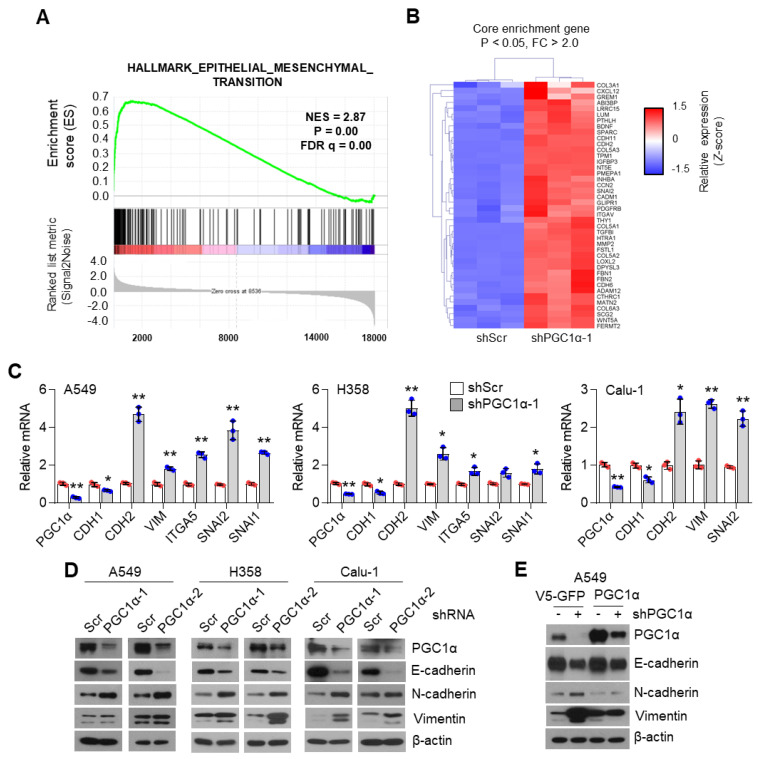

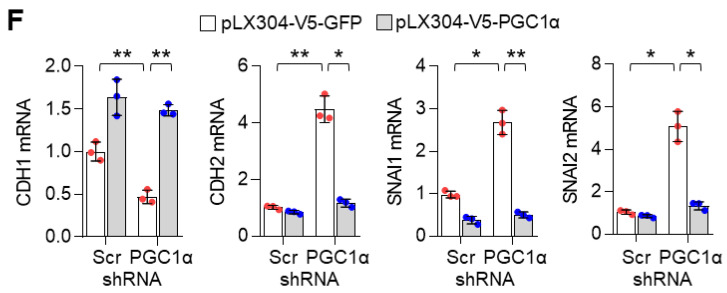
PGC1α loss promotes the epithelial–mesenchymal transition (EMT) in lung cancer cells. (**A**,**B**) Plot and heat map for the top gene set and core enrichment genes of the gene set enrichment analysis (GSEA) in A549 cells. (**C**) Expression of the EMT genes quantified by qPCR. (**D**) Western blots of the three EMT proteins and PGC1α in the control and PGC1α-silenced cells. (**E**) Western blots of indicated proteins in cells expressing GFP (pLX304-V5-GFP) or PGC1α (pLX304-V5-PGC1α) in the control and PGC1α knocked-down A549 cells. (**F**) qPCR of EMT genes. Values represent mean ± SD (*n* = 3). * *p* < 0.05 and ** *p* < 0.01 by Student’s *t*-test. The whole western blot images are showed in Figure S7.

To unravel the role of PGC1α in lung cancer, we first selected the ideal lung cancer cell lines, which showed either high or low levels of PGC1α, and we found that PGC1α levels are downregulated in lung cancer cells compared to Beas-2b, normal bronchial epithelial cells (Figure S1A,B). Short Tandem Repeat (STR) analysis was carried out to validate the lung cancer cell lines used in this study (Table S1). Because PGC1α levels in A549 cells were much higher than other lung cancer cell lines, we then analyzed the transcriptome to investigate the functional role of altered PGC1α expression in A549 lung cancer cells. An increased EMT gene expression signature was observed in PGC1α-silenced A549 cells (Figure 1A). We also compared the core enriched genes in the EMT gene set and found previously known EMT genes (Figure 1B and Figure S1C). Consistently, the suppression of PGC1α significantly decreased the expression of CDH1, an epithelial marker, whereas it increased the expression of CDH2, VIM, ITGA5, SNAI1, and SNAI2, which are the mesenchymal markers (Figure 1C). The alteration of EMT genes’ expression in PGC1α knocked-down lung cancer cells translated into changes in protein levels (Figure 1D). In addition, the restoration of PGC1α abolished the alteration of EMT genes and the expression of the corresponding proteins in PGC1α knocked-down A549 cells (Figure 1E,F). Collectively, these results support the speculation that the loss of PGC1α is required for EMT in lung cancer.

### 3.2. PGC1α Suppression Promotes Lung Cancer Initiation, Growth and Bone Metastasis

**Figure 2:**
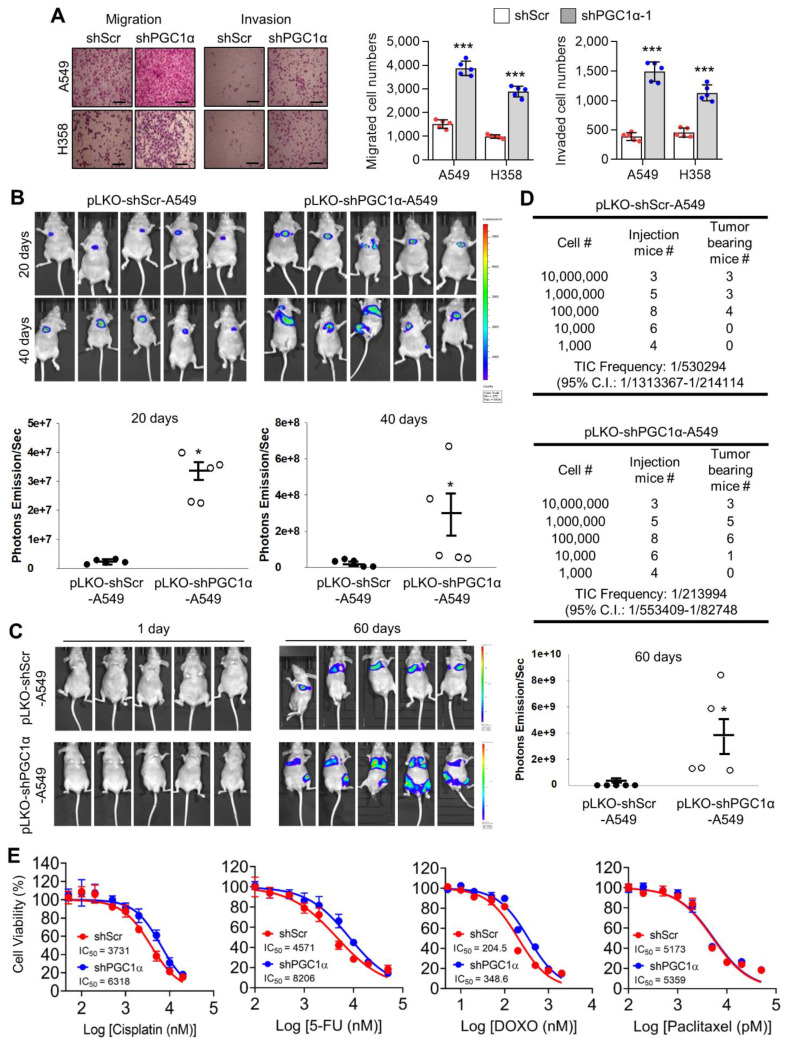
PGC1α loss promotes tumor initiation and lung cancer metastasis to bone. (**A**) Migrated and invaded cells in control and PGC1α-silenced A549 and H358 cells (*n* = 5). Scale bar represents 200 μm. High Resolution Images are showed in Figure S8. (**B**,**C**) Luciferase images from control or PGC1α-silenced A549 cells xenografted mice (*n* = 5) by intratracheal (**B**) and tail-vein (**C**) injection. Luciferase images represent one picture captured and the values of photons emission are represented as mean ± SEM of the indicated number of mice. * *p* < 0.05 by Student’s *t*-test. (**D**) In the serial dilution xenograft assays, a varying number (as indicated) of the control or PGC1α knocked-down A549 cells were transplanted in BALB/c-nude mice by subcutaneous (S.C.) injection and incubated for 4 weeks. (**E**) Cell viability analysis after the control or the PGC1α-silenced A549 cells were treated with cisplatin, 5-fluoruracil (5-FU), doxorubicin, and paclitaxel at different concentrations, as indicated, for 3 days. Values represent mean ± SD (*n* = 3). * *p* < 0.05 and *** *p* < 0.001 by Student’s *t*-test.

Because EMT associated with migration, invasion, chemoresistance, and tumor-initiating potential [2,35], we investigated whether PGC1α loss was responsible for lung cancer metastasis. PGC1α silencing promoted cellular motility and invasiveness in vitro (Figure 2A). To assess tumor growth and the metastatic properties of PGC1α suppression, an orthotopic metastasis model was utilized using PGC1α knocked-down A549 cells. In this assessment, we found that PGC1α suppression caused rapid tumor growth and led to several cases of bone metastasis (Figure 2B). PGC1α silencing caused a significant increase in the tumor growth in the lung and a reduction of mice survival rate (Figure S2A,B). Immunohistochemistry (IHC) analysis of orthotopic xenografts confirmed the changes in the EMT markers (Figure S2C). In line with these observations, we investigated whether PGC1α suppression promoted bone metastasis in lung cancer. We found that the silencing of PGC1α promoted bone metastasis in a tail-vein injection metastatic model (Figure 2C). To determine whether PGC1α suppression could enhance the tumor-initiating potential, we performed a subcutaneous xenograft by limiting dilution using PGC1α or control knocked-down A549 cells. Groups of mice were xenografted with several doses of A549 cells, over a range from a low dose insufficient to initiate tumor formation, to a high dose that certainly initiated tumor formation. Subsequently, we calculated that the tumor-initiating cells (TIC) frequency in PGC1α silencing were 59.7% upregulated more than that in control cells (Figure 2D), suggesting that PGC1α loss enhanced the tumor-initiating potential of lung cancer cells. In addition, PGC1α silencing showed chemo-resistance to cisplatin, 5-FU, and doxorubicin, but not to paclitaxel (Figure 2E). Consistent with this, an increase in the number of apoptotic cells induced by cisplatin was restored by the PGC1α suppression (Figure S2D). Together, these results reveal that the suppression of PGC1α expression could lead to tumor initiation, growth, and metastasis potential in lung cancer.

### 3.3. A Single Allele Deletion of Pgc1α Promotes Bone Metastasis of Kras^G12D^-Driven Lung Cancer

**Figure 3:**
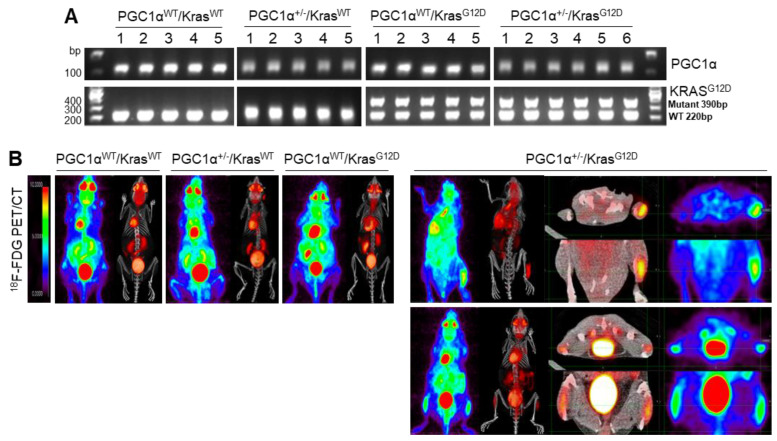

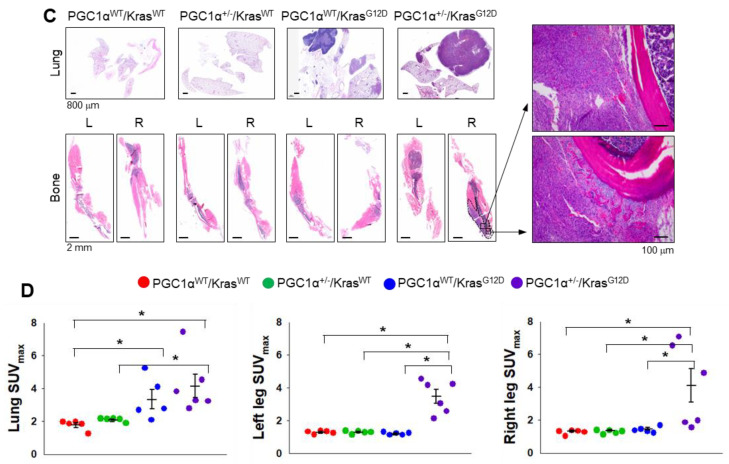
Bone metastasis of Kras^G12D^-driven lung cancer is promoted by a single Pgc1α allele knockout. (**A**) Genotyping PCR analysis of PGC1α^WT^; Kras^WT^, PGC1α^+/−^; Kras^WT^, PGC1α^WT^; Kras^G12D^, and PGC1α^+/−^; Kras^G12D^ mice are shown. The mice were selected from crossing heterozygous Tg Kras^G12D^ and heterozygous PGC1α^+/−^ mice based on the genotyping of the progeny. In accordance with a Mendelian trait, approximately 25% of the offspring exhibited in each group. (**B**) PET images of mice between 16 and 17 weeks of age. All images were normalized to the same maximal standard uptake value (SUVmax) to facilitate the comparison of PET lesions. Three-dimensional renderings of fused PET/CT images of the 5 mice from (**A**). (**C**) Hematoxylin and eosin (H&E)-stained tissue sections of lung (top) and leg (bottom) at the whole scan level and at a magnified level (right). In the H&E-stained tissue, tumors are circled in black. High Resolution Images are showed in Figure S8. (**D**) Quantification of tumor uptake value (SUVmax) and mean standardized uptake vales (SUVmax) of lung and legs. The whole western blot images are -shown in Figure S7. * *p* < 0.05 by Student’s *t*-test.

To examine the in vivo functions of PGC1α in spontaneous Kras^G12D^ mice model in terms of regulating tumorigenesis and metastasis, we generated mice with a heterozygous PGC1α whole body knockout (PGC1α^+/−^) (Figure S3). We then crossed PGC1α^+/−^ mice with transgenic mice that expressed an oncogenic gene, Kras^G12D^ (PGC1α^+/−^; Kras^G12D^). Transgenic and knockout mice above mentioned were confirmed by PCR genotyping (Figure 3A). As expected, Kras^G12D^ mice spontaneously developed multiple lung tumor nodules at the age of 4 months. The PGC1α^+/−^; Kras^G12D^ mice showed a significantly higher metastasis from lungs to bones, in particular to the right and left legs, than wild-type PGC1α, PGC1α^+/−^, and Kras^G12D^ mice (Figure 3B). Histological analysis showed increased bone metastasis in left and right legs (Figure 3C). An increased tumor burden in PGC1α^+/−^; Kras^G12D^ mice was also reflected by their SUVmax values compared with the age-matched wild-type PGC1α, PGC1α^+/−^, and Kras^G12D^ mice (Figure 3D). These results provide genetic evidence that the loss of PGC1α has a crucial role in mediating lung tumorigenesis and bone metastasis.

### 3.4. ID1 Is Required for the Loss of PGC1α-Mediated EMT

**Figure 4:**
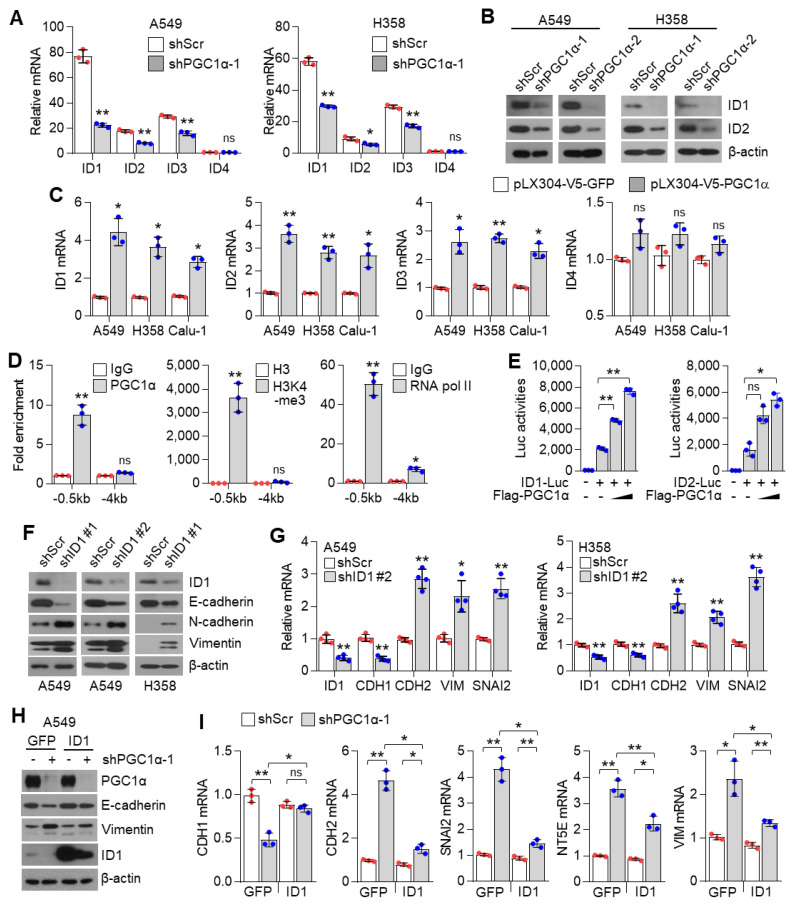
ID1 is a downstream target of PGC1α and is linked to EMT in lung cancer cells. (**A**) qPCR of ID genes in the control or PGC1α-silenced A549 and H358 cells (*n* = 3). (**B**) Protein expression of ID1 and ID2 in the control and PGC1α-silenced A549 and H358 cells. (**C**) Expression of the ID genes in GFP or PGC1α stably expressing lung cancer cells (*n* = 3). (**D**) ChIP for PGC1α, H3K4-me3, and RNA pol II followed by ChIP-qPCR in A549 cells confirms a significant binding of PGC1α at the ID1 proximal and distal promoter loci (−0.5 kb and −4 kb) (*n* = 3). (**E**) ID1 (−289 bp) and ID2 (−352 bp) promoter luciferase analysis. Luciferase vector (200 ng/mL) was transiently transfected into HEK293 cells with or without Flag-PGC1α (50, 100, 200 ng/mL) (*n* = 3). (**F**) Western blots of indicated proteins in the control or the ID1 knocked-down A549 and H358 cells. (**G**) Expression of the EMT genes in the control and ID1 silenced cells (*n* = 3). (**H**) Western blots of indicated proteins in cells stably expressing GFP (pLX304-V5-GFP) or ID1 (pLX304-V5-ID1) in the control and the PGC1α knocked-down A549 cells. (**I**) EMT genes expression in cells expressing GFP or ID1 in the control and the PGC1α knocked-down A549 cells (*n* = 3). ns, not significant; * *p* < 0.05 and ** *p* < 0.01 by Student’s *t*-test. The whole western blot images are showed in Figure S7.

The inhibitor of DNA binding 2 (ID2) is an essential component during melanoma metastasis promoted by PGC1α suppression [12]. Thus, we investigated whether ID proteins were responsible for promoting EMT induced by PGC1α silencing. Surprisingly, we found that mRNA and protein levels of ID1, ID2, ID3, but not of ID4, were downregulated by PGC1α knock-down (Figure 4A,B and Figure S4A); however, ID1, ID2, and ID3 were increased by PGC1α overexpression (Figure 4C). Thus, perhaps, the key question is—which one, among ID1, ID2, and ID3, predominantly functions during EMT mediated by PGC1α suppression? To answer this question, we analyzed the relative expression of IDs in multiple types of cancer cell lines. Our results showed a higher and more dominant expression of ID1 than those of ID2, ID3, and ID4, particularly in lung cancer cell lines (Figure S4B). The Cancer Genome Atlas (TCGA) lung adenocarcinoma (LUAD) and lung squamous cell carcinoma (LUSC) RNA-sequencing samples presented similar results (Figure S4C). To investigate whether PGC1α bound to the active promoter region of the ID1 gene, chromatin immunoprecipitation (ChIP) was performed. The active promoter regions with H3K4-me3, H3K27-ac and RNA polymerase II on the ID gene are delineated in Figure S4D. We found that PGC1α occupied the proximal promoter region (−0.5 kb) of the ID1 gene along with H3K4-me3 and RNA pol II (Figure 4D), suggesting that PGC1α is an essential transcriptional component for regulating ID1 expression. Ectopic expression of PGC1α increased ID1 and ID2-promotor luciferase activity (Figure 4E). We then examined the effect of ID1 repression in the context of EMT, using EMT genes and proteins and found similar results as those observed in PGC1α-silenced lung cancer cells (Figure 4F,G). Furthermore, restoration of ID1 reversed the expression of EMT markers altered by PGC1α loss (Figure 4H,I). Collectively, these results indicate that ID1 is required for the EMT process upon PGC1α suppression in lung cancer cells.

### 3.5. PGC1α and ID1 Is Decreased in Lung Cancer and Associated with a Poor Clinical Outcome

**Figure 5:**
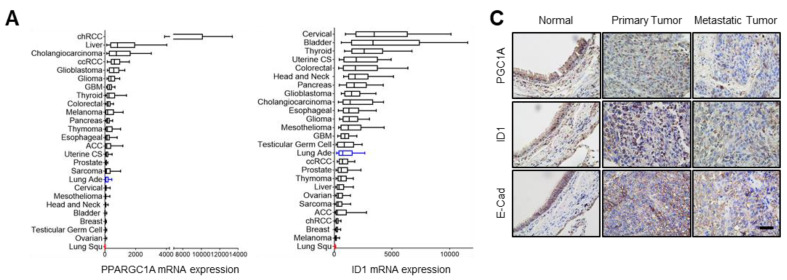

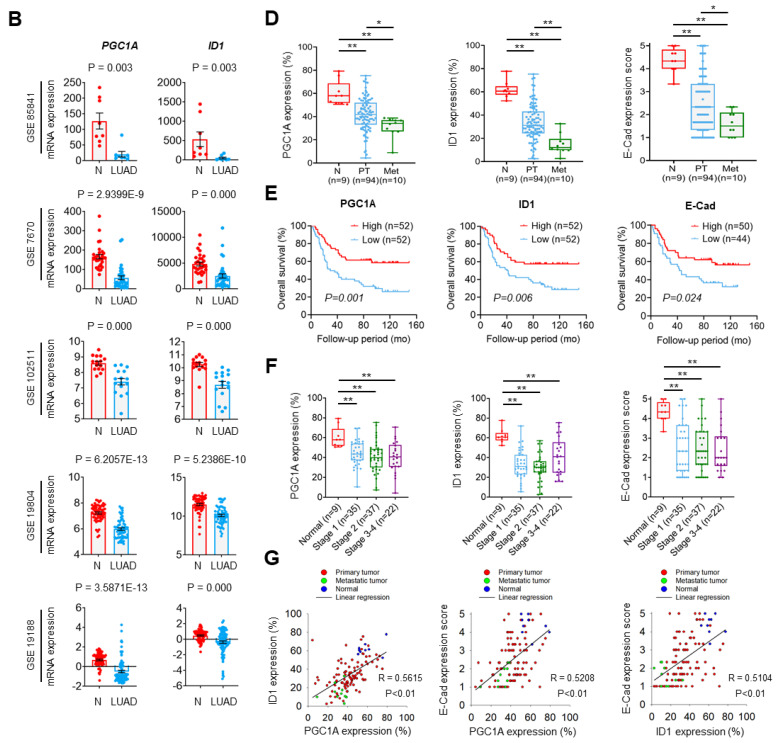
PGC1α and ID1 are downregulated in lung cancers and associated with a poor prognosis of lung cancer patients. (**A**) The mRNA expressions of PPARGC1A (PGC1α) or ID1 in 26 human cancer tissues were evaluated by using RNA Seq V2 RSEM. The box plots show the distribution of values and the middle lines within boxes show the mean values. All data were downloaded from cBioportal (www.cbioportal.org (accessed on 1 January 2020). (**B**) PGC1α and ID1 mRNA expression levels in up to five additional lung cancer datasets (N, normal; LUAD, lung adenocarcinoma). Sample sizes: GSE85841 (N, 8; LUAD, 8); GSE7670 (N, 30; LUAD, 35); GSE102511 (N, 15; LUAD, 16); GSE19804 (N, 60; LUAD, 60); GSE19188 (N, 65; LUAD, 91). (**C**) IHC staining of PGC1α, ID1, and E-cadherin in normal and lung cancer tissues. Bar, 50 μm. High Resolution Images are showed in Figure S8. (**D**) The expression of PGC1α, ID1, and E-cadherin in normal lung (N), primary tumor (PT), and metastatic (Met) specimens in lung tissues. Each dot represents one patient specimen. (**E**) Association of the indicated proteins with Kaplan–Meir overall survival (OS) analysis in lung cancer patients. Sky blue and red lines indicate low- and high-expression groups of the indicated proteins, respectively. (**F**) Protein levels of PGC1α, ID1, and E-cadherin according to tumor stages in normal lung and lung cancer tissues. (**G**) Correlation between PGC1α and ID1 or PGC1α and E-cadherin, or ID1 and E-cadherin. Correlation values (R) were determined by spearman’s correlation test. The horizontal lines in all plots indicate the mean ± SE. *p* value was calculated by Mann–Whitney U-test (**B**,**D**,**F**), and the log-rank test (**E**) respectively. * *p* < 0.05 and ** *p* < 0.01.

To investigate the role of the PGC1α-ID1 axis in human lung cancer on a large scale, we firstly examined PGC1α and ID1 mRNA expressions in 26 different major cancers using TCGA database. Consistently with our findings, PGC1α and ID1 is expressed at a low level in lung cancer (Figure 5A). In other types of cancers, such as breast and ovarian cancer, no change in the expression of PGC1α and ID1 was observed between normal tissues and cancer tissues (Figure S5A,B). We extended these findings to five additional datasets in which gene expression datasets for normal and LUAD are publicly available. A decrease in the expression of PGC1α and ID1 mRNAs in LUAD compared with normal tissues was observed in all datasets (Figure 5B). To evaluate the correlation between PGC1α, ID1, and E-cadherin expressions at the protein level, we performed IHC in human lung cancer tissue arrays. The patients’ clinical information is summarized in Table S2. We detected that the expression of PGC1α, ID1, and E-cadherin was progressively reduced from primary tumors to metastatic tissues (Figure 5C,D). When the lung cancer tissues were sub-divided into PGC1α_high and PGC1α_low groups using a median value of PGC1α, a lower overall survival rate was evident in the PGC1α_low compared with the PGC1α_high (Figure 5E). Similar results were observed regarding ID1 and E-cadherin (Figure 5E). However, the levels of PGC1α, ID1, and E-cadherin in lung cancers did not decrease significantly with the tumor stage (Figure 5F). PGC1α expression revealed a strong correlation with ID1 in lung cancer tissues. Furthermore, PGC1α and ID1 expression positively correlated with the expression of E-cadherin, the representative epithelial marker (Figure 5G). These results suggest that the expression of PGC1α and ID1 was downregulated in lung cancers, with an expression pattern reminiscent of a high metastatic potential and a poor clinical outcome.

### 3.6. TCF4 Promotes TWIST1-Mediated EMT

**Figure 6:**
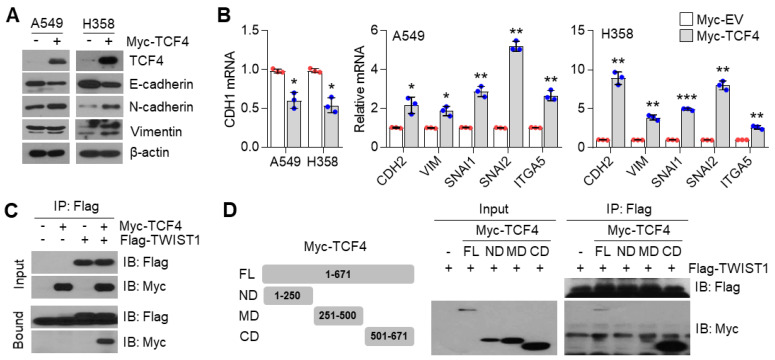

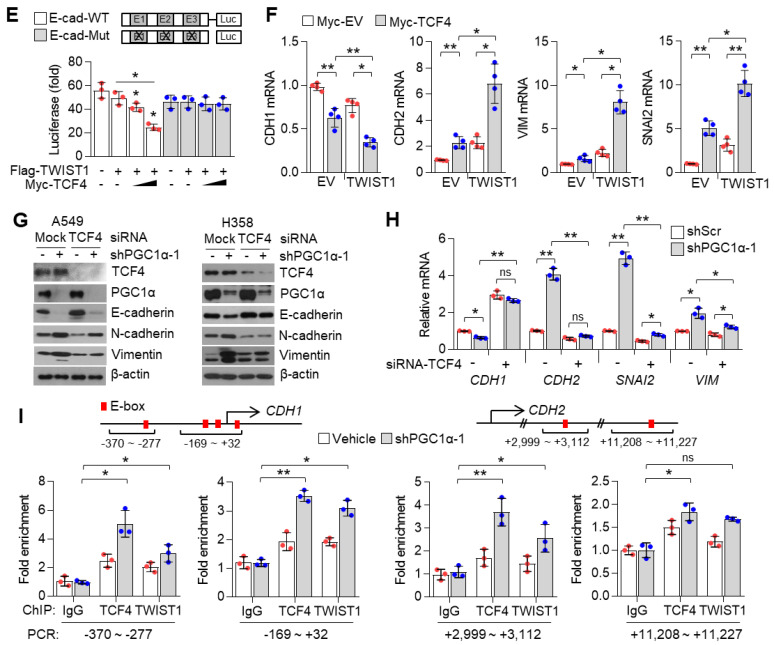
TCF4 promotes EMT by cooperating with TWIST1. (**A**) Western blots of indicated proteins. (**B**) Expression of the EMT genes (*n* = 3). (**C**) Interaction of TCF4 and TWIST1. (**D**) Binding domain of TCF4 and TWIST1. Myc-TCF4 and its mutants (FL, Full length; ND, N-terminal domain; MD, Middle domain; CD, C-terminal domain) were transfected into HEK293T cells with Flag-TWIST1 as indicated, and the interaction between TCF4 and TWIST1 was analyzed using co-immunoprecipitation and Western blotting. (**E**) E-cadherin-wild type and -mutant promoter luciferase analysis (*n* = 3). (**F**) Expression of the EMT genes in cells expressing pLX304-V5-GFP or pLX304-V5-TWIST1 in the control and Myc-TCF4 transfected A549 cells (*n* = 4). (**G**) Western blots of indicated proteins. (**H**) EMT genes expression in A549 cells (*n* = 3). (**I**) ChIP for TCF4, TWIST1, and IgG, followed by ChIP-qPCR in the control or in PGC1α-silenced A549 cells confirms the binding of TCF4 and TWIST1 on the CDH1 or CDH2 promoter loci (*n* = 3). Values represent mean ± SD. ns, not significant; * *p* < 0.05, ** *p* < 0.01, and *** *p* < 0.001 by Student’s *t*-test. The whole western blot images are showed in Figure S7.

How does the PGC1α-ID1 axis regulate EMT? A previous report has shown that TCF4, an E-box binding transcription factor (E-protein), closely participates in the wide spread of melanoma in response to PGC1α suppression [12]. In addition, upregulated TCF4 levels have been observed in a highly bone-metastatic subpopulation of H460 lung cancer cells [36]. Thus, we speculated that TCF4 is a potential effector upon PGC1α-ID1 axis-mediated EMT and generated stable A549 cells overexpressing TCF4 to investigate whether TCF4 was responsible for the EMT by PGC1α loss. Ectopic expression of TCF4 decreased the expression of CDH1, whereas it was found to increase VIM, SNAI1, SNAI2, ITGA5, and CDH2, indicating that TCF4 regulates the EMT (Figure 6A,B). We observed a positive correlation between TCF4 and known-EMT markers (ETS1, MMP2, SNAI2, VIM, ZEB1, ZEB2, FN1) from the TCGA database (Figure S6A). Lung cancer cell lines presented a higher expression of TCF4 compared to breast cancer cell lines (Figure S6B). To understand the precise molecular mechanism by which TCF4 regulated the EMT, we speculated that TWIST1, which contains bHLH domain and acted as an EMT promoting factor, might cooperate with TCF4. As speculated, TWIST1 was found to interact with TCF4 (Figure 6C), particularly at the carboxyl-terminal domain (a.a 501-671) of TCF4 (Figure 6D). Does TCF4 act as a transcriptional activator for TWIST1 on the EMT? To answer this question, the CDH1 promoter reporter (which contains TWIST1 binding E-box) assay was performed. In this assay, TCF4 was found to enhance the suppressive effect of TWIST1 on CDH1 promoter reporter (Figure 6E). Similar to this, when TCF4 was ectopically co-expressed with TWIST1, an additional decrease in CDH1 and an increase in the CDH2, VIM, and SNAI2 was observed, when compared with the ectopic expression of TCF4 alone (Figure 6F), thereby suggesting that TCF4 acts as a transcriptional activator of TWIST1. Similarly, it was observed that TCF4 knock-down abolished the alteration of EMT markers in PGC1α-silenced cells (Figure 6G,H). Next, we investigated whether TCF4 cooperates with TWIST1 to regulate EMT gene expression at the chromatin levels. We found that TCF4 was enriched on the CDH1 and CDH2 promoter region with TWIST1, and this enrichment was significantly increased by PGC1α knock-down (Figure 6I). These results indicate that the cooperation between TCF4 and TWIST1 is responsible for the loss of PGC1α-induced EMT.

### 3.7. ID1 Attenuates EMT by Interfering with the TCF4-TWIST1 Interaction

**Figure 7:**
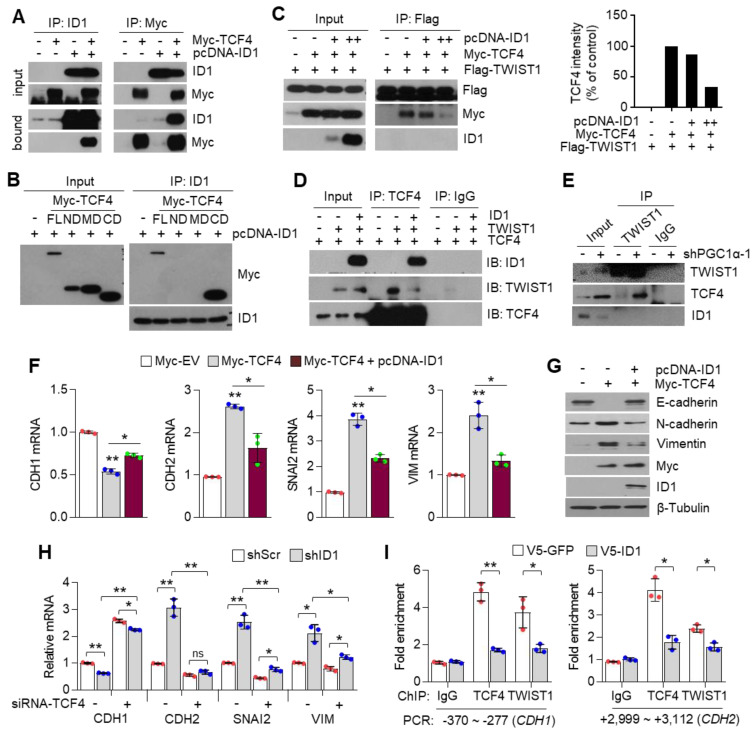

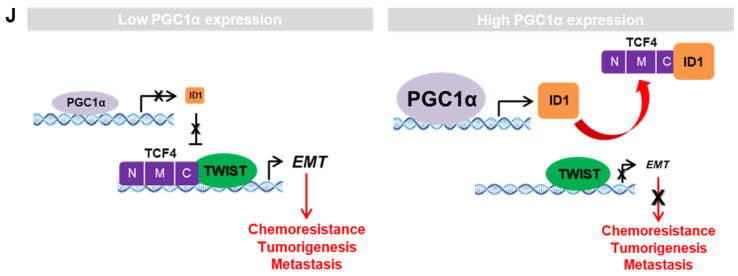
ID1 inhibits the interaction and transcriptional activity of TCF4 and TWIST1. (**A**) The protein–protein interaction of ID1 and TCF4. Interaction between TCF4 and ID1 was analyzed using co-immunoprecipitation and Western blotting. (**B**) ID1 interacts with the C-terminal region of TCF4. (**C**) Interaction between TCF4 and TWIST1 upon ectopic ID1 expression was analyzed by co-immunoprecipitation and Western blotting. The levels of TCF4 protein, which binds to TWIST1, was quantified by using image J. (**D**) In vitro protein–protein interaction analysis using recombinant proteins. Interaction between TCF4 and TWIST1 was measured by co-immunoprecipitation using anti-TCF4 or anti-IgG antibodies and Western blotting. (**E**) Endogenous TCF4 and TWIST1 interaction in PGC1α-silenced A549 cells. (**F**,**G**) Expression of the EMT genes (**F**) and proteins (**G**) in A549 cells expressing Myc-EV or Myc-TCF4 with or without pcDNA-ID1 as indicated (*n* = 3). (**H**) Expression of the EMT genes in A549 cells (*n* = 3). (**I**) ChIP for TCF4, TWIST1, and IgG followed by ChIP-qPCR in PGC1α knocked-down A549 cells transfected with GFP and ID1 (*n* = 3). (**J**) Graphical summary of the proposed molecular mechanism by which decreased PGC1α suppresses ID1, and consequently increases TCF4-TWIST1-mediated EMT. Conversely, high levels of PGC1α expression-induced ID1 result in suppression of EMT by interfering the interaction between TCF4-TWIST1. Black and red arrows represent the positive effect, ┴ line represents the negative effect, and X represents the suppressive effect on the proposed working model. Values represent mean ± SD. ns, not significant; * *p* < 0.05 and ** *p* < 0.01 by Student’s *t*-test. The whole western blot images are showed in Figure S7.

ID1 is an endogenous inhibitor of E-protein, and consequently dissociates the interaction between E-protein and bHLH transcription factor on the E-box containing promoter [30]. In addition, ID1 is predominantly expressed in lung cancer cells and is directly regulated by PGC1α at the transcriptional level (Figure 4). Thus, we speculated whether ID1 acted as an inhibitor of TCF4 and TWIST1 during EMT induced by PGC1α loss. It was observed that ID1 strongly interacts with TCF4 (Figure 7A), and this interaction occurs particularly in the c-terminal domain of TCF4, where it shares an interaction with TWIST1 (Figure 7B). Consistent with the mechanistic function of ID1, the interaction between TCF4 and TWIST1 was disrupted by the ectopic expression of ID1 (Figure 7C). This interfering role of ID1 on TCF4 and TWIST1 interaction was confirmed using an in vitro co-immunoprecipitation assay for each recombinant protein (Figure 7D). Furthermore, the increased endogenous interaction of TCF4 and TWIST1, caused by the down-regulation of ID1, was observed in PGC1α knocked-down cells (Figure 7E). The CDH1 promoter reporter exhibited that the suppression of CDH1 promoter activity by the TCF4 and TWIST1 co-expression was restored by ID1. The mutated CDH1 reporter did not respond to TCF4 or ID1 (Figure S6C). Further, ID1 was found to reverse the TCF4-mediated down-regulation of CDH1 and the up-regulation of CDH2, SNAI2, and VIM (Figure 7F,G). Conversely, TCF4 silencing reversed the changes of EMT markers expression, which were induced by ID1 suppression alone (Figure 7H). We observed that ectopic expression of ID1 attenuated the PGC1α silencing-induced recruitment of TCF4 and TWIST1 on promoter region of CDH1 and CDH2 genes (Figure 7I). These results indicate that ID1 acts as an endogenous inhibitor of TCF4-TWIST1-mediated EMT.

## 4. Discussion

PGC1α modifies the cellular metabolism in cancer cells and influences cancer development and progression [8,9,11,12]. A decreased PGC1α has been reported to promote lung metastasis from subcutaneous primary melanoma [12]. Similarly, PGC1α suppresses prostate cancer aggressiveness and metastasis by activating the estrogen-related receptor alpha (ERRα)-dependent transcriptional program [9] or inhibiting polyamine synthesis [37]. PGC1α promotes colorectal tumorigenesis and tumor growth via de novo lipogenesis [8]. In breast cancers, PGC1α increases cell migration and invasion, and facilitates metastasis [11]. Additionally, PGC1α expression is considered a characteristic feature of cancer stem cells in pancreatic cancers, possibly imparting chemoresistance to anti-cancer drugs [38]. Therefore, the functional role of PGC1α regarding tumor initiation, chemoresistance, and metastasis might differ depending on the cancer type. In the present study, orthotopic and subcutaneous xenograft analysis using PGC1α knocked-down A549 cells showed that PGC1α loss enhanced the tumor initiating and metastatic potential of lung cancer. Furthermore, the spontaneous lung cancer model using Kras^G12D^ transgenic and PGC1α knockout mice showed that PGC1α loss promoted Kras^G12D^-driven lung cancer growth and metastasis, in particular, to bone.

During metastasis, cancer cells of epithelial origin often reprogram their cells to a mesenchymal type, in a process termed epithelial–mesenchymal transition (EMT) that helps to spread them to other organs, via transcriptional programming, which is regulated by TWIST1, ZEB1, SLUG, and SNAIL [39]. In the present study, transcriptome and gene expression analysis showed that PGC1α loss significantly regulated EMT-related gene expression. As an epithelial marker, CDH1, was decreased and mesenchymal markers, CDH2, VIM, SNAI2, and ITGA5 were increased in PGC1α-silenced epithelial types of A549, H358, and Calu-1 lung cancer cells. Thus, our results indicate that PGC1α loss enhances the tumor-initiating and metastatic potential of lung cancer by activating EMT. Considering that EMT-activating transcription factors (EMT-TFs) are linked to chemoresistance [40], we showed that chemoresistance to anti-cancer drugs such as cisplatin, 5-FU, and doxorubicin, might be attributed to the loss of PGC1α-induced EMT.

ID family members contribute to tumor development, cell differentiation, proliferation, and angiogenesis, and thus, are considered as tumor promoting factors that facilitate tumor growth and metastasis [32]. ID1 has been especially associated with tumor progression and metastasis and is well-studied in breast cancer. The invasion and metastatic potential of breast cancer have been closely associated with ID1 expression and are needed for the maintenance of cancer stem-like features [41,42]. Likewise, an overexpression of ID1 has been detected in malignant small cell lung cancer (SCLC) cell lines compared to normal Beas-2b cells [43] and showed a poor prognosis of NSCLC patients [44], and induced cell proliferation and metastasis in NSCLC [45]. We also found that ID1 is highly expressed in lung cancers among other ID proteins, which was confirmed in vitro, by using bioinformatics and analysis via the publicly available TCGA dataset; thus, these results led us to hypothesize that ID1 played a crucial role in PGC1α loss-induced EMT. Although previous studies support the oncogenic role of ID1, we revealed the cancer-suppressor function of ID1 in lung cancer, which was regulated by PGC1α. Notably, ID1 expression is lost in most of metastatic human lung adenocarcinoma specimens. Further, a decreased expression of ID1 is positively correlated with mesenchymal markers, while restoration of ID1 showed epithelial features. In line with our results, overexpression of ID1 has been reported in the cytoplasm, while its low expression is reported in the nuclei of human SCLC biopsy specimens [43]. Consistently with our study, the suppressive role of ID1 on EMT by antagonizing TCF3 (E2A), which belongs to bHLH (basic helix–loop–helix) containing E-protein, has been identified [46]. Based on our results, we suggest that ID1 is predominantly involved in EMT in lung cancer metastasis and speculate that the differential expression levels of ID1 between the cytoplasm and the nuclei might distinguish between the oncogenic or tumor suppressive role of ID1.

Although the contribution of the PGC1α-ID2 axis in melanoma metastasis has been well studied [12], the mechanisms underlying the regulation of ID families by PGC1α is poorly understood in lung cancer. We found that PGC1α not only promotes ID1, but also ID2 and ID3 expression in lung cancer cells, suggesting that PGC1α-mediated expression of ID families might be dependent on the cellular context and transcription factors, which are activated by PGC1α. Specifically, chromatin immunoprecipitation (ChIP) and promoter reporter assay showed that PGC1α binds to the proximal promoter region (−0.5 kb) of ID1 and activates the promoter reporter containing the proximal promoter region of ID1 and ID2, indicating that PGC1α regulates ID1 and ID2 at the transcriptional level. In addition, 3 out of 26 major cancers (breast, ovarian, and lung cancers) expressed PGC1α and ID1 at a lower level in the TCGA dataset. Among those, one out of three cancers (Lung cancer) consistently showed an altered expression of PGC1α and ID1 throughout databases. IHC results also revealed the low expression of PGC1α and ID1 in primary lung cancer (PLC). This expression was hardly detected in metastatic lung cancer tissues. This is, to the best of our knowledge, the first report that shows that PGC1α is closely associated with ID1 expression in lung cancer. In addition, our data show that PGC1α protein expression in cultured lung cancer cells are lower than Beas-2b, normal bronchial epithelial cells. Interestingly, similar expression of PGC1α in A549, H1666, and Beas-2b were observed, indicating that A549 and H1666 cells express basal levels of PGC1α, and these are suitable to investigate the pro-metastatic effects of PGC1α loss. Despite the lack of related literature, a few reports support our findings. A previous report showed the direct interaction between ID1 and PGC1α regulates thermogenesis in brown adipose tissue (BAT) [47]. Since ID1 is predominantly expressed in lung cancer among the ID family, we reasoned that ID1 serves as an important factor in the loss of PGC1α-driven EMT and propose that PGC1α acts as a transcriptional regulator of ID1 expression.

TWIST1 belongs to the class II bHLH family and heterodimerizes with the ubiquitously expressed class I bHLH members, known as E-proteins [48]. TWIST1, EMT-promoting transcription factor, is an essential for cancer metastasis [49]. A recent report revealed that the TWIST1 homodimer and heterodimer interactions with E-proteins, such as TCF3/E2A, TCF4/ITF-2, and TCF12/HEB, enhances EMT [50]. Among those, the class I bHLH factor, TCF4, is one of the last additions among the EMT regulating factors, and acts as an E-cadherin repressor by interacting with other molecules. Qin et al. showed that TWIST1 induced cancer progression by using TCF4 as a co-regulatory protein [49]. TWIST1 and TCF4 protein interaction was also validated in osteosarcoma cells [51]. Based on these reports, we speculated that TCF4 was a potential binding partner and transcriptional co-activator of TWIST1 on EMT gene expression. Indeed, our data showed that TWIST1 binds to the c-terminal region of TCF4, and our results were validated by in vitro and in vivo studies. TCF4 enhances TWIST1-mediated CDH1 suppression and mesenchymal markers expression. Which tumor microenvironment is associated with PGC1α loss-mediated EMT? TGFβ1 is a major inducer of EMT through transcriptional reprogramming with SNAI1, SNAI2, ZEB1, and ZEB2 as well as TWIST1 [7]. Recent report has shown that PGC1α suppresses TGFβ1/Smad signaling through *let-7b/c* upregulation [52]. In addition, suppression of ID1 and ID2 expression by TGFβ1/Smad signaling has been previously observed [53,54]. Thus, we guess that TGFβ1/Smad signaling could be associated with PGC1α to finetune ID1-TCF4-TWIST1 transcriptional axis regulating EMT and lung cancer metastasis.

In the process of bone metastasis in lung, breast, and prostate cancer, tumor-driven osteoclastogenic factors, such as TGFβ1, PTHLH (parathyroid hormone-like hormone), and MMP-2 (Matrix metalloproteinase-2), facilitate osteolysis and homing of cancer cells to bone [55,56]. In addition, Vicent et al. have reported that TCF4 is upregulated in the highly metastatic subpopulation of H460 lung cancer cells that invade bones [36]. Interestingly, our transcriptome and gene expression analysis shows that PGC1α loss increases PTHLH and MMP-2. Consistently, bone metastasis from lung cancer was observed in xenograft and GEM models, indicating that PGC1α loss resulting in the activation of TCF4-TWIST1 might be associated with lung cancer bone metastasis, and with the transcriptional reprogramming of the expression of osteoclastogenic factors.

ID proteins function as dominant-negative regulators of bHLH transcription factors (such as E-proteins family), in this case, of TCF4, through the formation of non-functional heterodimers, and therefore block their DNA binding and transactivation of their target genes [57]. Various studies have revealed the association of E-proteins with their negative regulators (ID family) in the development of diseases such as Rett syndrome (TCF3, ID1-4), atherosclerosis (TCF3, ID3), Diamond Blackfan anemia (TCF12, ID2), polycystic kidney disease (TCF3, ID2), and Burkitt’s lymphoma (TCF3, ID3) [58,59,60,61,62]. Similarly, our study revealed that ID1 directly interacts with the c-terminal region of TCF4 and weakens the binding affinity of TCF4 to the target gene promoter, leading to the attenuation of the transcription activity of TCF4-regulated genes. As ID1 and TWIST1 share the binding domain of TCF4, PGC1α knock down-driven ID1 loss could not interfere with the TCF4-TWIST1 complex, and thus, induced EMT potential in the process of lung cancer metastasis.

## 5. Conclusions

We found that PGC1α functions as a tumor suppressor of the TCF4-TWIST1-induced EMT transcriptional circuit in lung cancer malignancy via increasing ID1 expression, which can competitively bind to TCF4. This loop is broken with the loss of PGC1α expression, which leads to lung cancer metastasis. The major findings include: (1) Decreased PGC1α and ID1 levels were observed in human lung primary and metastatic tumor specimens and were closely associated with a poor prognosis; (2) the suppression of PGC1α and ID1 promoted tumor growth, tumor-initiating potential, and bone metastasis through EMT in lung cancer; (3) the interaction between TCF4 and TWIST1, which is inhibited by ID1, was responsible for PGC1α loss-induced EMT. Taken together, these findings allow us to propose that targeting the components downstream of PGC1α could provide diagnostic and therapeutic targets for lung cancer patients.

## Data Availability

For all data requests, please contact the corresponding author. RNA-seq data and analysis are deposited to GEO (GSE156833).

## Supplementary Materials

The following are available online at https://www.mdpi.com/article/10.3390/cancers13081772/s1, Figure S1: Suppression of PGC1α upregulates EMT-related genes expression in lung cancer cells, Figure S2: Suppression of PGC1α causes chemoresistance and metastasis, Figure S3: Targeting Ppargc1α (Pgc1α) allele and generation of knockout mice, Figure S4: ID1 is a target of PGC1α and predominantly expressed in lung cancer, Figure S5: PGC1α and ID1 mRNA expression is not altered in ovarian and breast cancer biopsies, Figure S6: TCF4 mRNA is correlated with EMT-related genes expression, Figure S7: Whole western blot images, Figure S8: High Resolution Images of Figure 2A, Figure 3C and Figure 5C, Table S1: Confirmation of lung cancer cell lines by using STR analysis, Table S2: Clinical Information for Human Lung Cancer Patients, Table S3: Oligonucleotide sequences for short hairpin RNA, qRT-PCR, ChIP-PCR and gene cloning, Table S4: Antibodies information for western blotting, chromatin immunoprecipitation (ChIP) and immunoprecipitation, and uncropped whole scan of western blots film.
