# Supplementary material for: PGC1α Loss Promotes Lung Cancer Metastasis through Epithelial-Mesenchymal Transition
Source: Cancers (Basel). 2021 Apr 8;13(8):1772. doi: 10.3390/cancers13081772 (PMC8068195; doi:10.3390/cancers13081772)
Supplement: Supplementary file 1 [file cancers-13-01772-s001.pdf]

cancers

Article

PGC1α Loss Promotes Lung Cancer Metastasis through Epithelial-Mesenchymal Transition

Taek-In Oh [1,2,†], Mingyu Lee [3,4,5,†], Yoon-Mi Lee [1,†], Geon-Hee Kim [1,2], Daekee Lee [6], Jueng Soo You [7], Sun Ha Kim [8], Minyoung Choi [8], Hyonchol Jang [8], Yeong-Min Park [9], Hyun-Woo Shin [3,4,5,*], Dong Hoon Shin [8,*] and Ji-Hong Lim [1,2,10,*]

1 Department of Biomedical Chemistry, College of Biomedical & Health Science, Konkuk University, Chungju 27478, Korea; dk1050@kku.ac.kr (T.-I.O.); yoonmilee@kku.ac.kr (Y.-M.L.); rlarjsgml4@kku.ac.kr (G.-H.K.)
2 Department of Applied Life Science, Graduate School, BK21 Program, Konkuk University, Chungju 27478, Korea
3 Obstructive Upper airway Research (OUaR) Laboratory, Department of Pharmacology, Seoul National University College of Medicine, Seoul 03080, Korea; alligator08@snu.ac.kr
4 Department of Biomedical Sciences, Seoul National University Graduate School, Seoul 03080, Korea
5 Cancer Research Institute, Seoul National University College of Medicine, Seoul 03080, Korea
6 Department of Life Science, Ewha Womans University, Seoul 03760, Korea; daekee@ewha.ac.kr
7 Department of Biochemistry, School of Medicine, Konkuk University, Seoul 05029, Korea; jsyou@kku.ac.kr
8 Research Institute, National Cancer Center & Department of Cancer Biomedical Science, National Cancer Center Graduate School of Cancer Science and Policy, Goyang 10408, Korea; 1905205@ncc.re.kr (S.H.K.); cmy413@ncc.re.kr (M.C.); hjang@ncc.re.kr (H.J.)
9 Department of Immunology, School of Medicine, Konkuk University, Seoul 05029, Korea; immun3023@kku.ac.kr
10 Diabetes and Bio-Research Center, Konkuk University, Chungju 27478, Korea
* Correspondence: charlie@snu.ac.kr (H.-W.S.); dhshin@ncc.re.kr (D.H.S.); jhlim@kku.ac.kr (J.-H.L.); Tel.: +82-2-740-8285 (H.-W.S.); +82-31-920-2499 (D.H.S.); +82-43-840-3567 (J.-H.L.)
† These authors contributed equally.

Citation: Oh, T.-I.; Lee, M.; Lee, Y.-M.; Kim, G.-H.; Lee, D.; You, J.S.; Kim, S.H.; Choi, M.; Jang, H.; Park, Y.-M.; et al. PGC1α Loss Promotes Lung Cancer Metastasis through Epithelial-Mesenchymal Transition. *Cancers* **2021**, *13*, x. https://doi.org/10.3390/xxxxx

Academic Editor: Daniel Neureiter

Received: 7 March 2021
Accepted: 30 March 2021
Published: date

Publisher's Note: MDPI stays neutral with regard to jurisdictional claims in published maps and institutional affiliations.

Copyright: © 2021 by the authors. Submitted for possible open access publication under the terms and conditions of the Creative Commons Attribution (CC BY) license (http://creativecommons.org/licenses/by/4.0/).

Supplementary

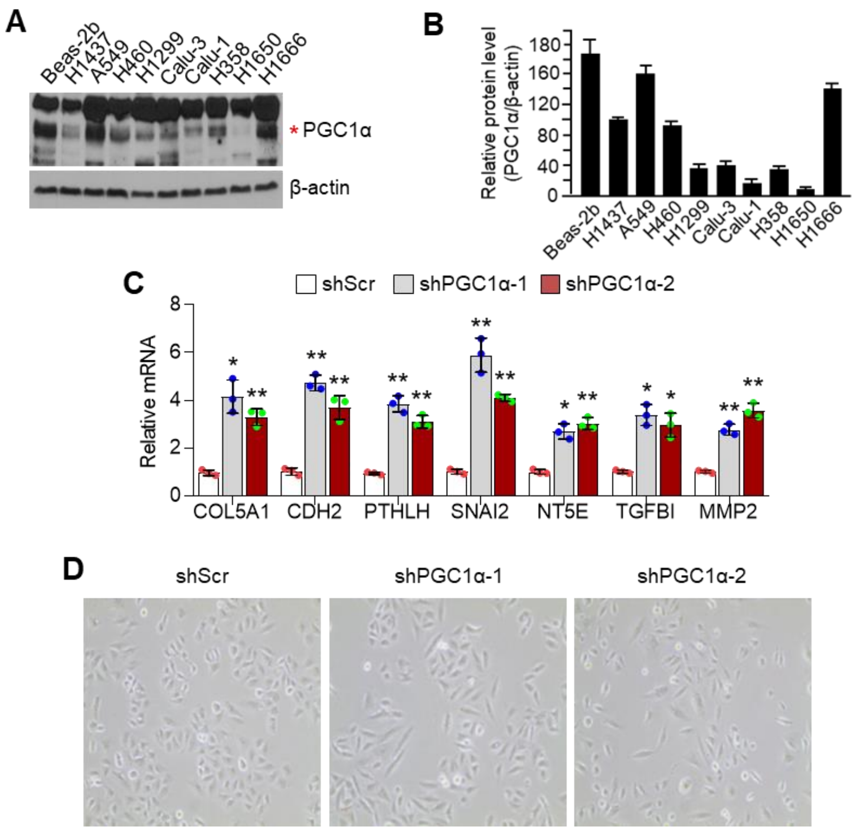

Figure S1. Suppression of PGC1α upregulates EMT-related genes expression in lung cancer cells. (A) Lung cancer cell lines were collected for western blot analysis. The red asterisk denotes PGC1α. (B) The PGC1α protein level was normalized to β-actin level. (C) The mRNA expression associated with epithelial and mesenchymal markers in the control or PGC1α silenced A549 cells. (D) Morphological change in control or PGC1α knock-down A549 cells. Values are represented as mean ± SD (n=3). *p < 0.05 and **p < 0.01 by Student's t-test.

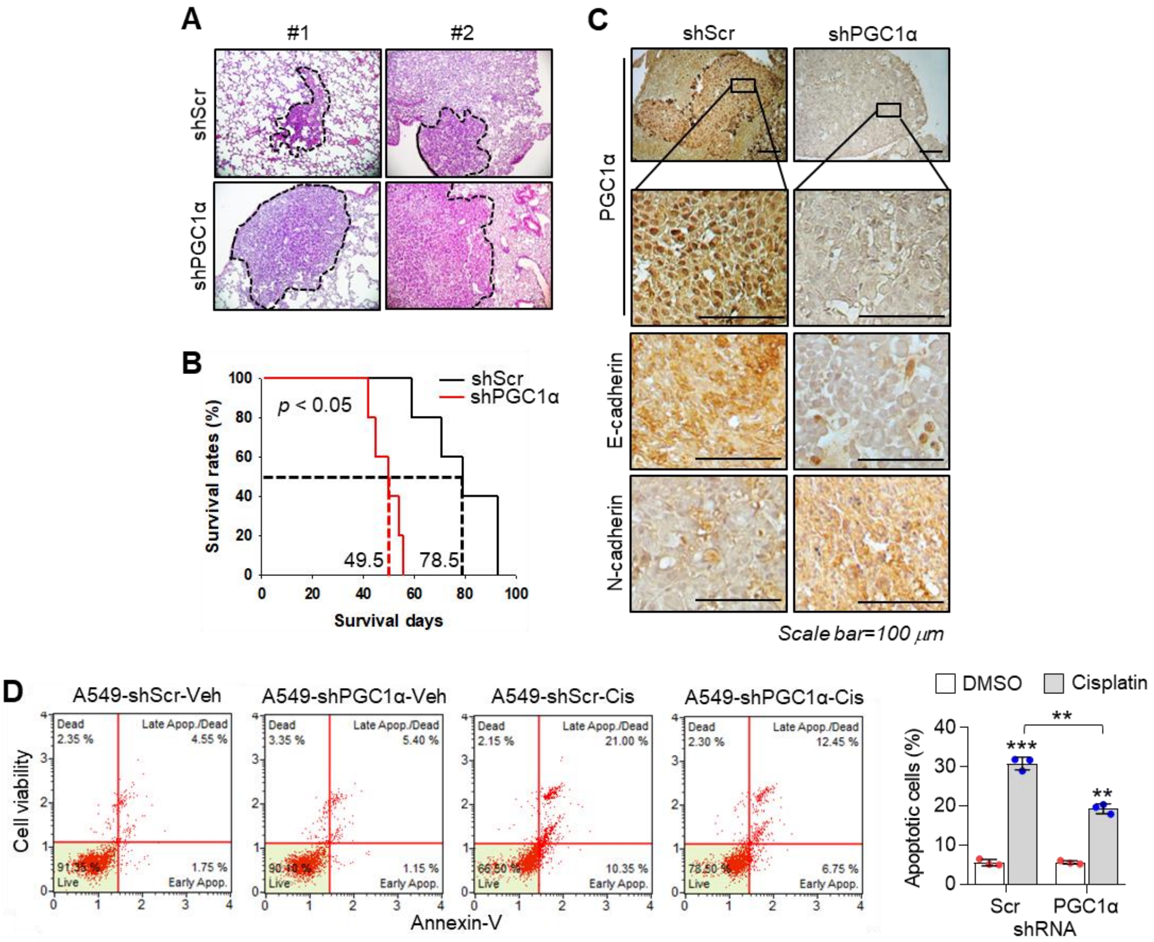

Figure S2. Suppression of PGC1α causes chemoresistance and distant metastasis. (A) Histopathological analysis of xenografted tumors in lung tissue for hematoxylin and eosin (H&E) staining. (B) The Kaplan-Meier survival curves in mice with lung cancer xenografts by intratracheal (I.T.) injection. Survival rates in the A549 xenograft groups are represented and statistically analyzed using log-rank test (n=5). (C) Representative images of immunohistochemistry (IHC) for PGC1α, E-cadherin, and N-cadherin in xenografted primary tumor tissues. Rectangle boxes indicate magnified areas. (D) Annexin-V staining analysis and quantified apoptotic cell numbers in the control or in PGC1α knocked-down A549 cells treated with cisplatin (5 μM) for 3 days. Values are represented as mean ± SD (n=3). **$p < 0.01$ and ***$p < 0.001$ by Student's t-test.

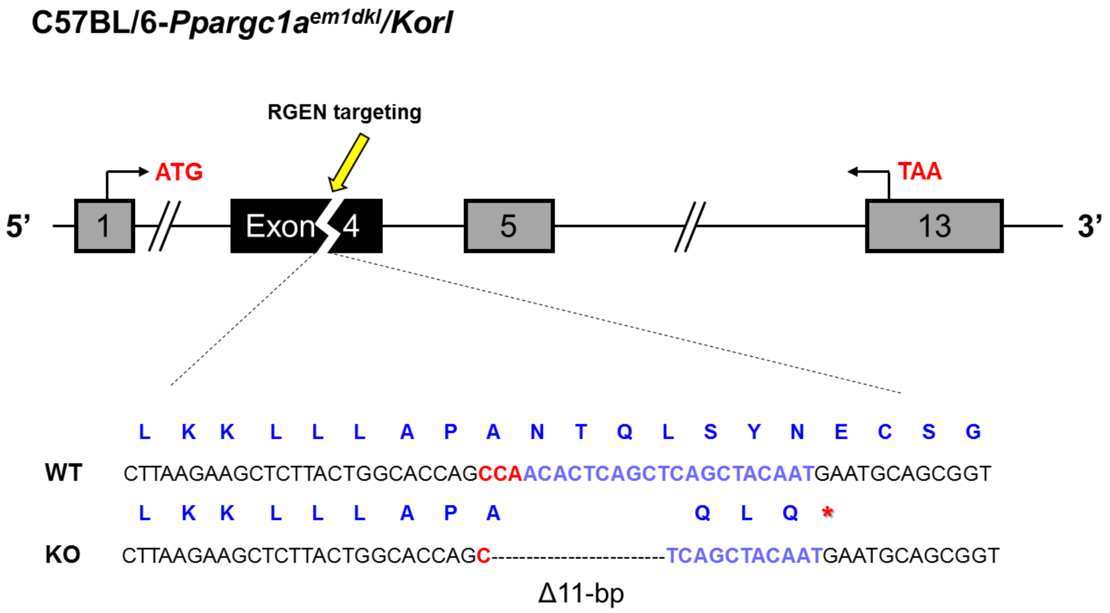

Figure S3. Targeting *Ppargc1α* (*Pgc1α*) allele and generation of knockout mice. Schematic representation of Ppargc1α (Pgc1α) knockout mice generation by targeting the Pgc1α exon 2.

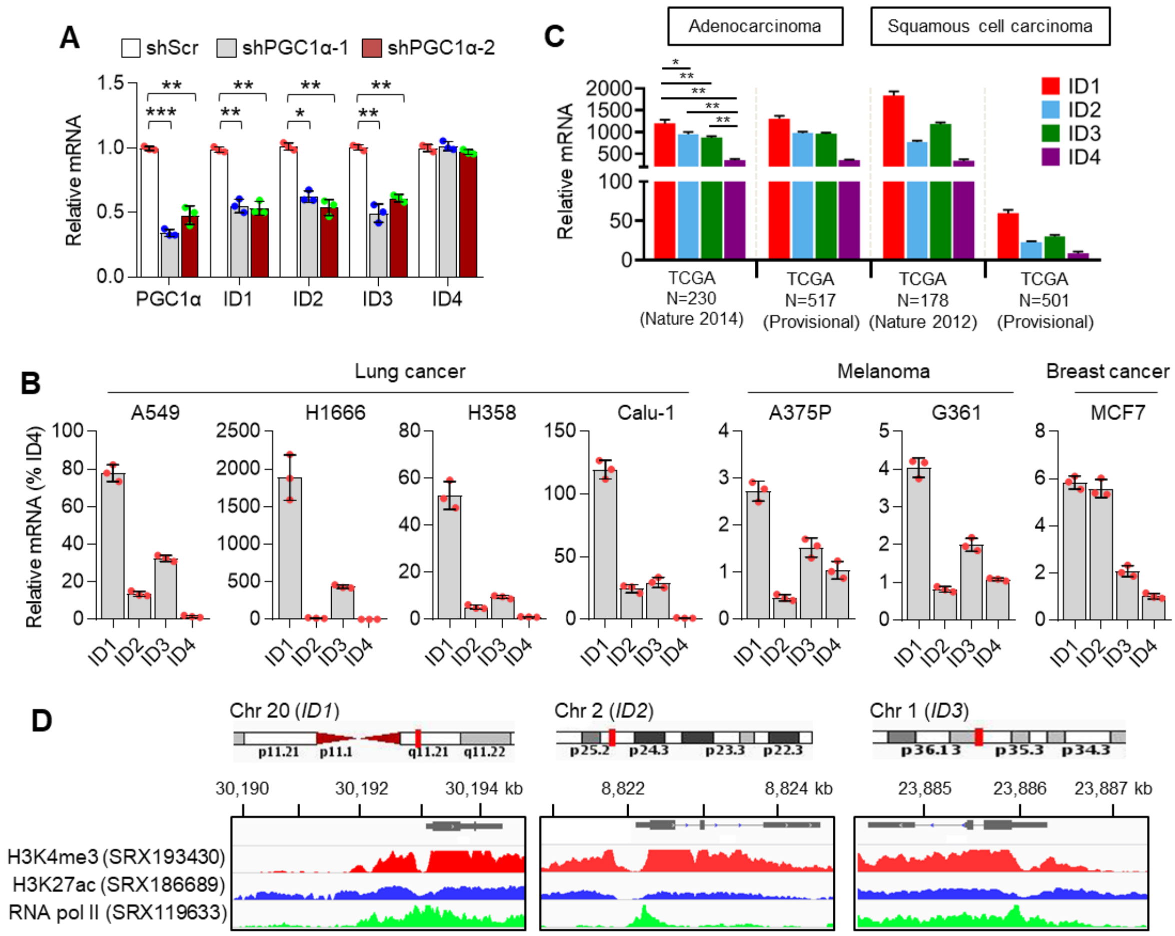

Figure S4. ID1 is a target of PGC1α and predominantly expressed in lung cancer. (A) Relative mRNA expression of IDs in the control or in PGC1α knocked-down A549 cells. Values are represented as mean ± SD (n=3). *$p < 0.05$, **$p < 0.01$, and ***$p < 0.001$ by Student's t-test. (B) Relative mRNA expression of IDs in lung (A549, H1666, H358, and Calu-1), melanoma (A375P and G361), and breast (MCF7) cancer cell lines, as indicated. Values are represented as mean ± SD (n=3). (C) IDs mRNA expression levels in lung cancer data sets from the cancer genome atlas (TCGA), as indicated. *$p < 0.05$ and **p <

0.01 by Mann-Whitney U-test. (D) ChIP-seq profiles of histone markers and RNA polymerase II enrichment indicating the transcriptionally active region of ID1, ID2, and ID3. Representative images obtained by ChIP-Atlas (https://chip-atlas.org/) and integrative genomic viewer (IGV).

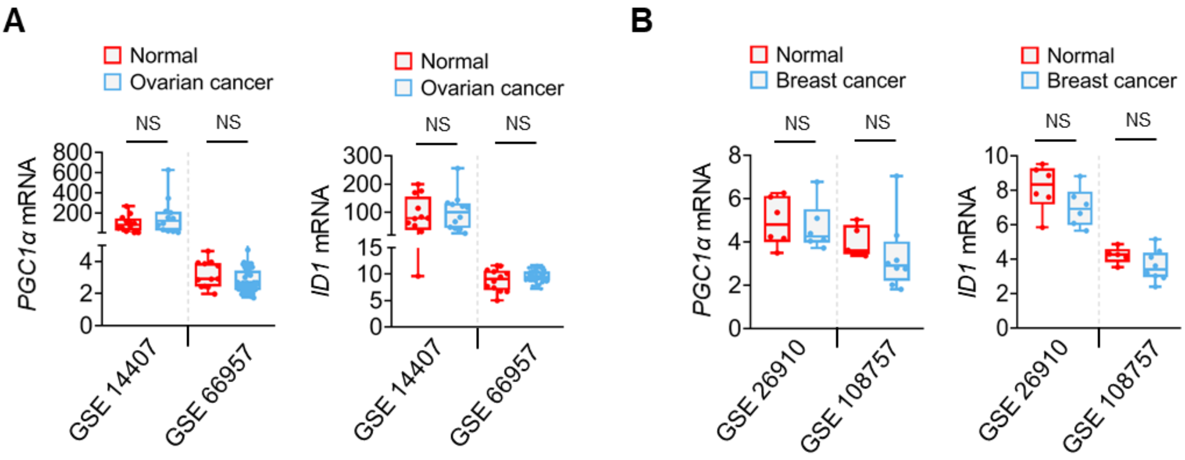

Figure S5. PGC1α and ID1 mRNA expression is not altered in ovarian and breast cancer biopsies. (A) The mRNA levels of PGC1α and ID1 in ovarian cancer and (B) breast cancer data sets, respectively. Sample sizes: GSE14407 (N, 12; ovarian cancer, 12); GSE66957 (N, 12; ovarian cancer, 57); GSE26910 (N, 6; breast cancer, 6); GSE108757 (N, 5; breast cancer, 8). Each bar represents the mean ± SE. The statistical significance was calculated by Mann-Whitney U-test. NS, not significant.

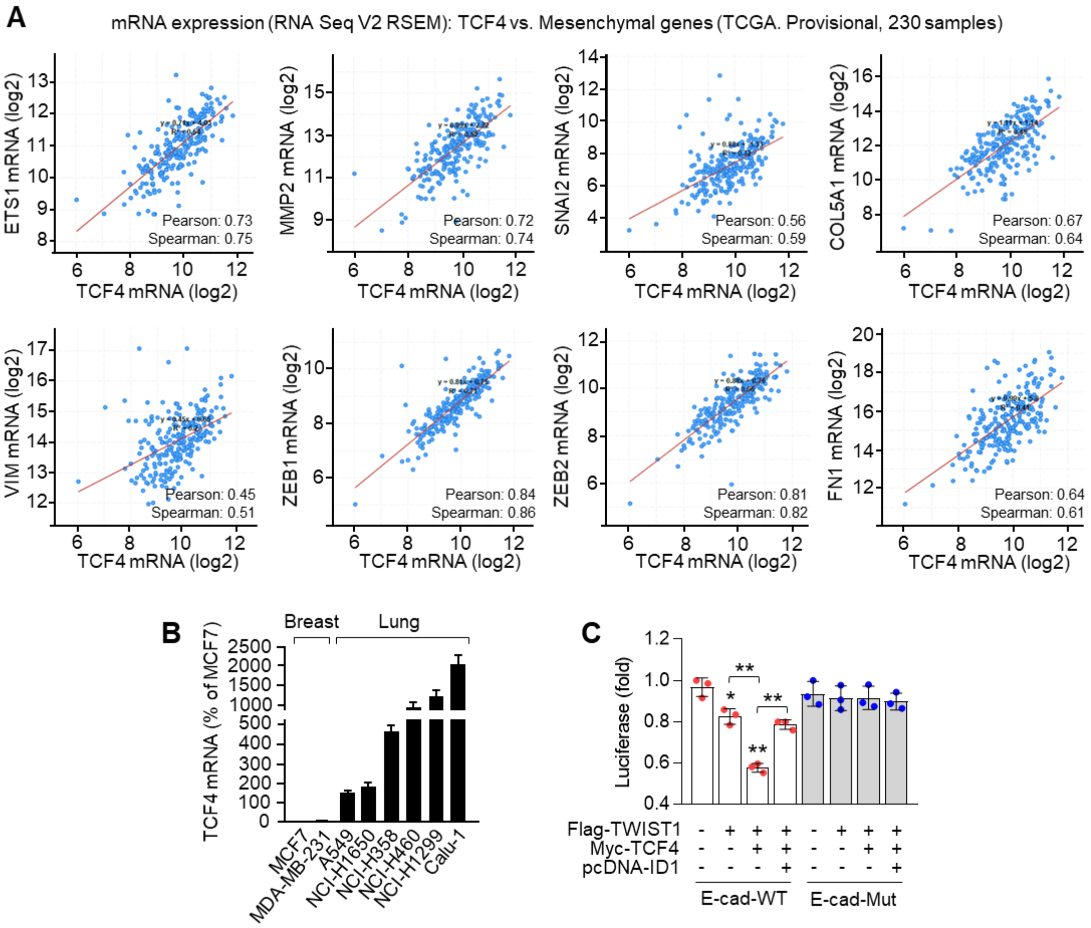

Figure S6. TCF4 mRNA is correlated with EMT-related genes expression. (A) Gene expression data from human cancers was analyzed using the cBioportal for cancer genomics and correlation plots between TCF4 and mesenchymal markers with Spearman's correlations are represented. (B) TCF4 mRNA levels in lung and breast cancer cell lines, as indicated.

Values are represented as mean ± SD (n=3). (C) E-cadherin-WT and E-cadherin-Mut luciferase vectors were transfected into HEK293T cells with or without Flag-TWIST1, Myc-TCF4, and pcDNA-ID1, as indicated. Values are represented as mean ± SD (n=3). *p < 0.05 and **p < 0.01 by Student's *t*-test.

Figure 1D

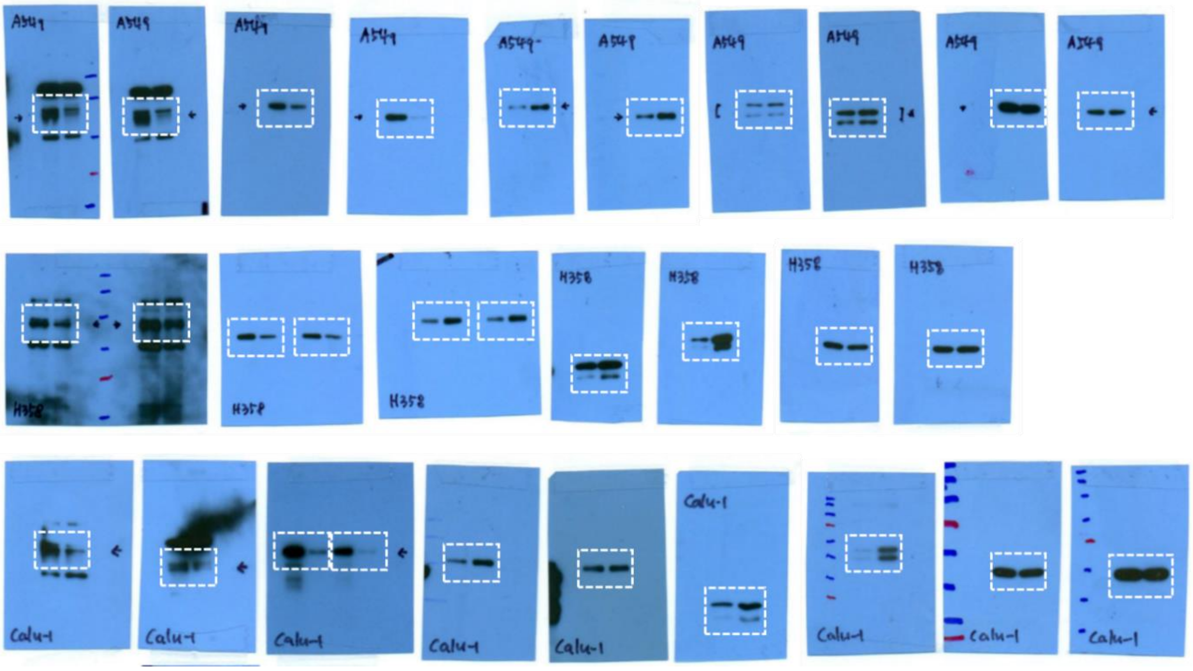

Figure 1E

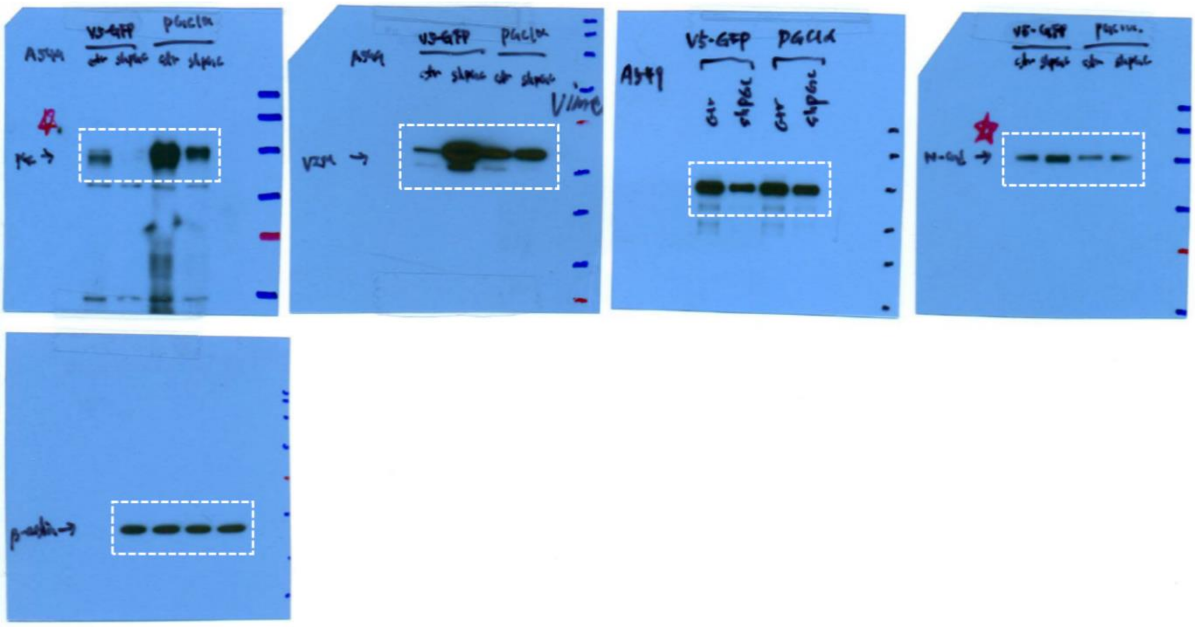

Figure 4B

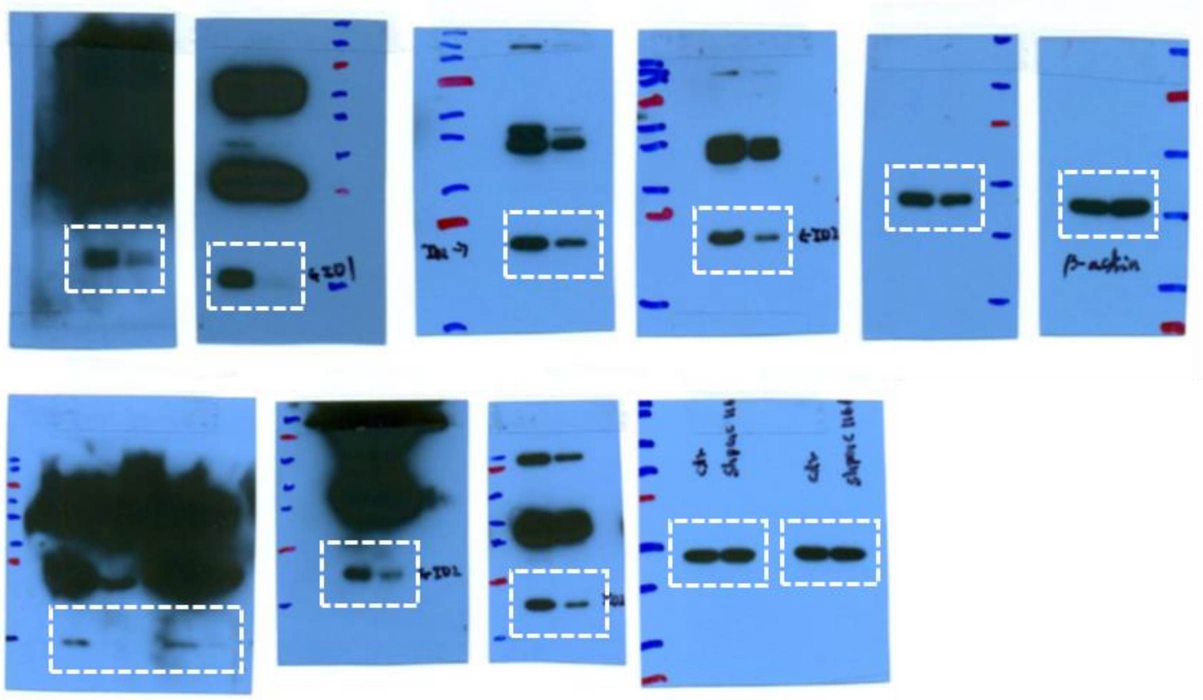

Figure 4F

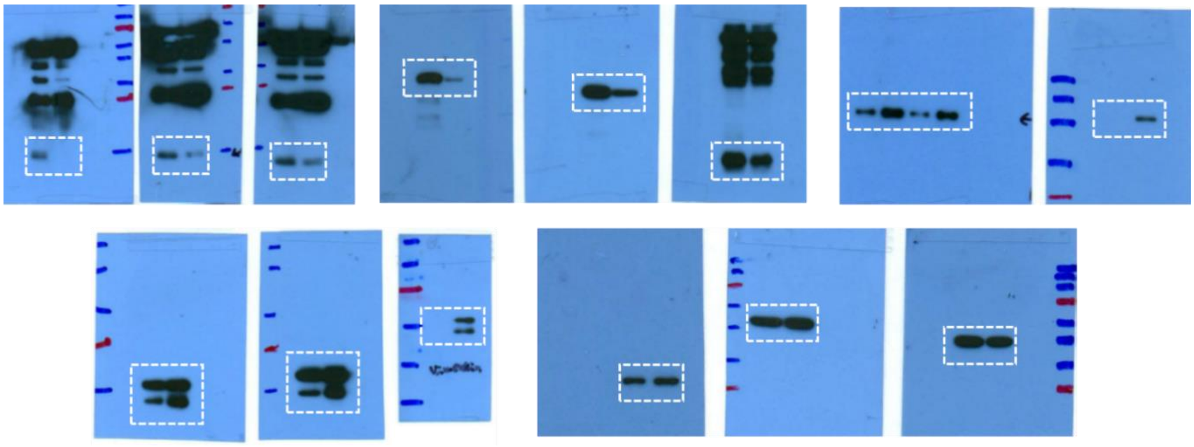

Figure 4H

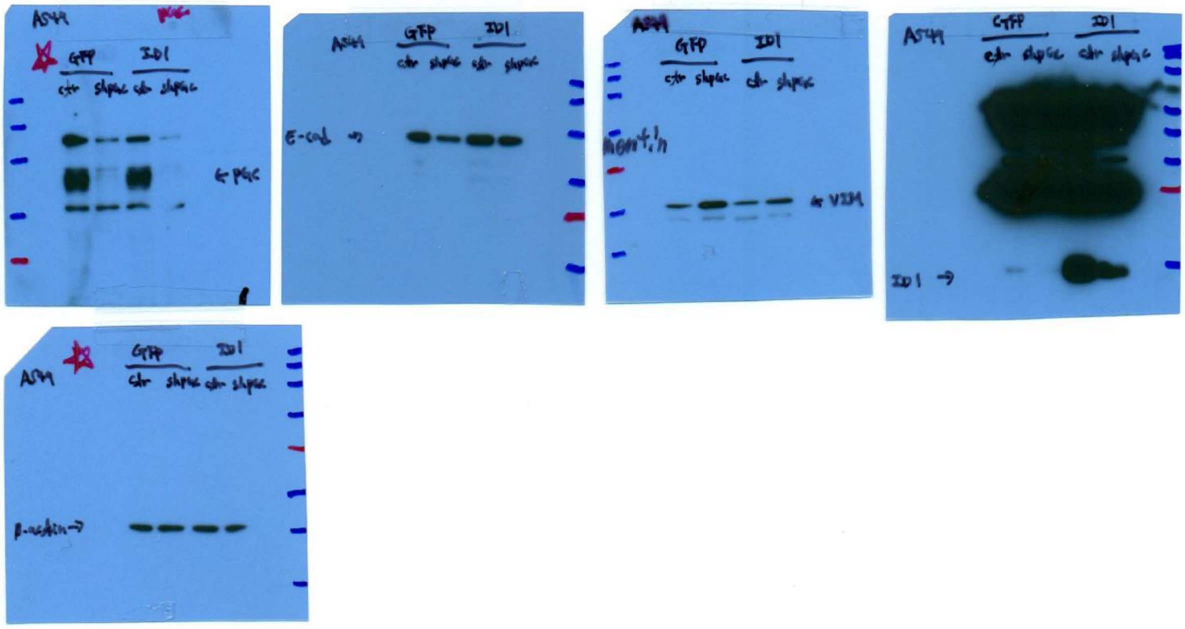

Figure 6A

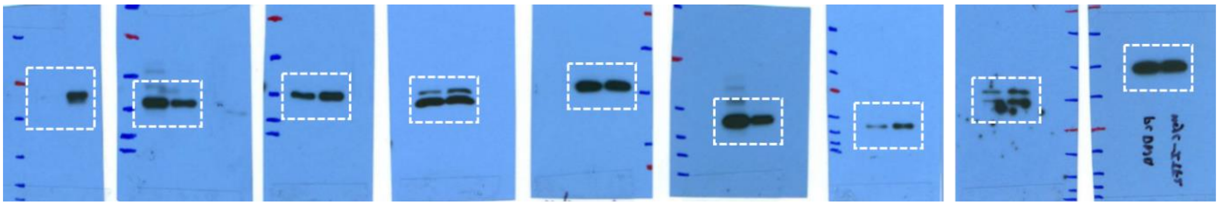

Figure 6C

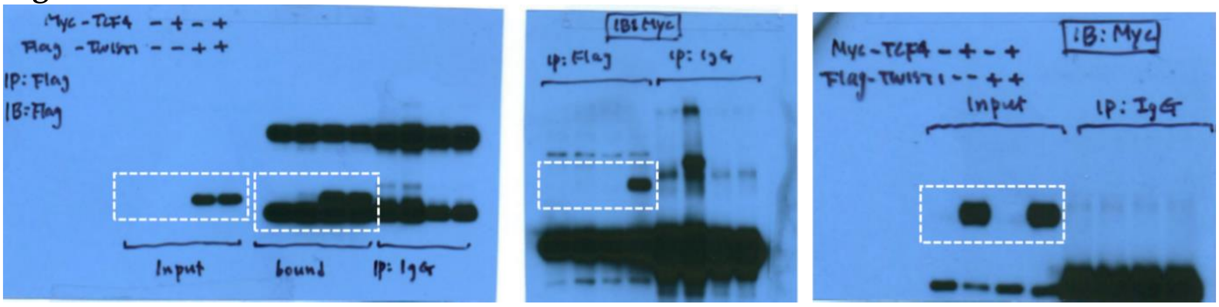

Figure 6D

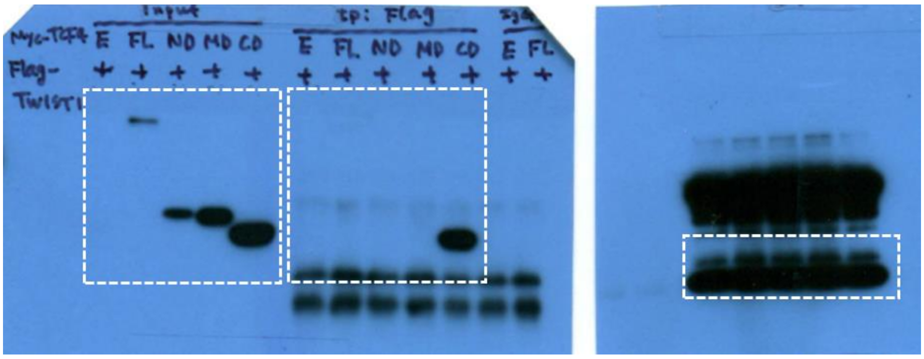

Figure 6G

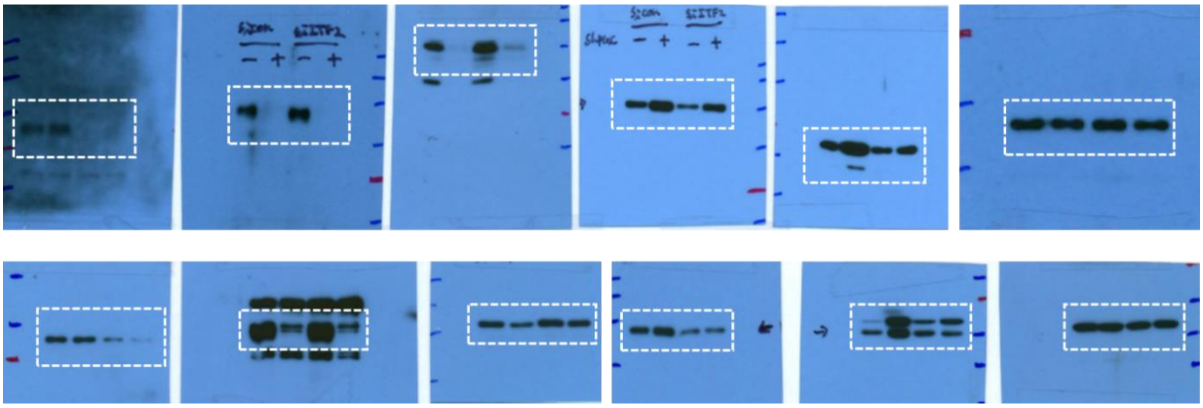

Figure 7A

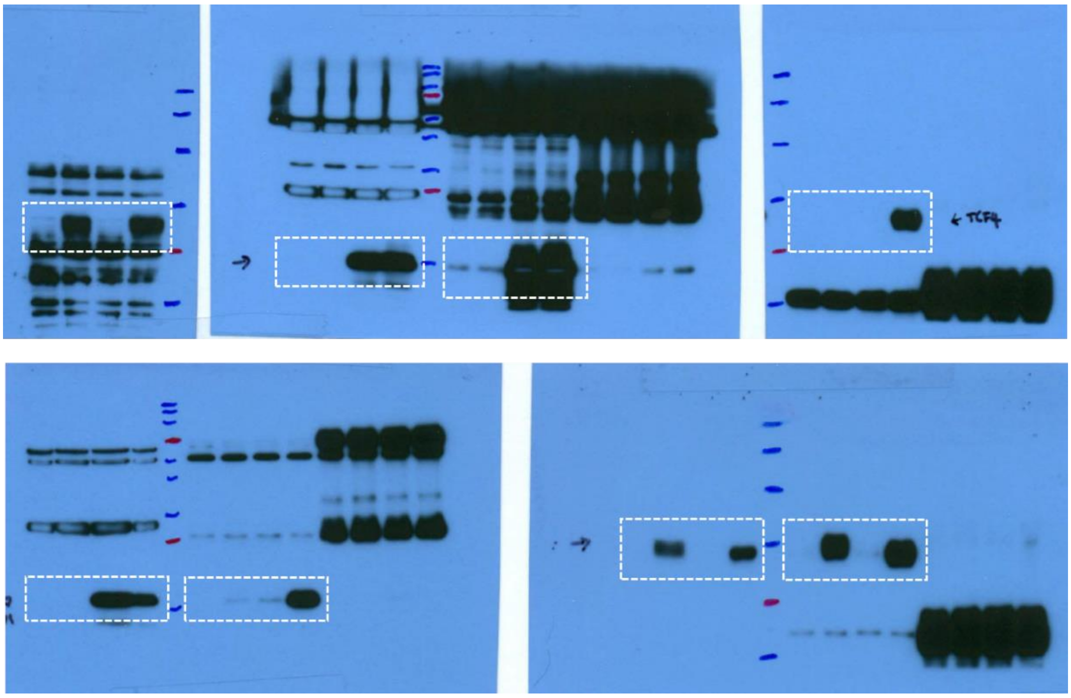

Figure 7B

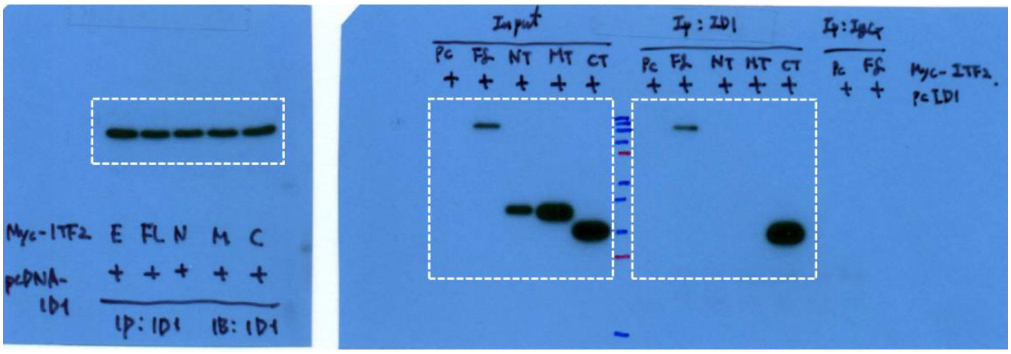

Figure 7C

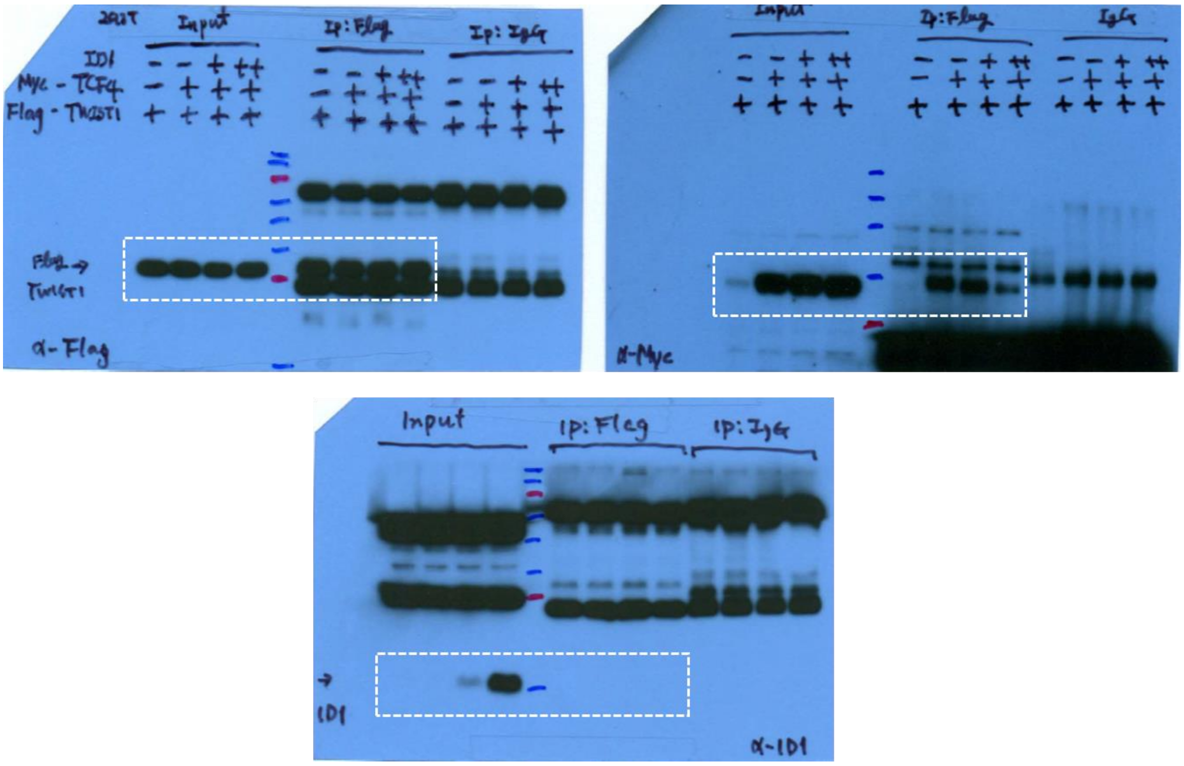

Figure 7D

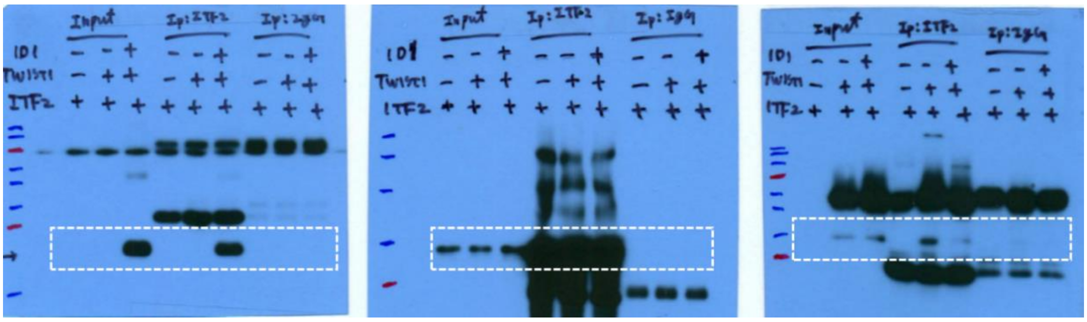

Figure 7E

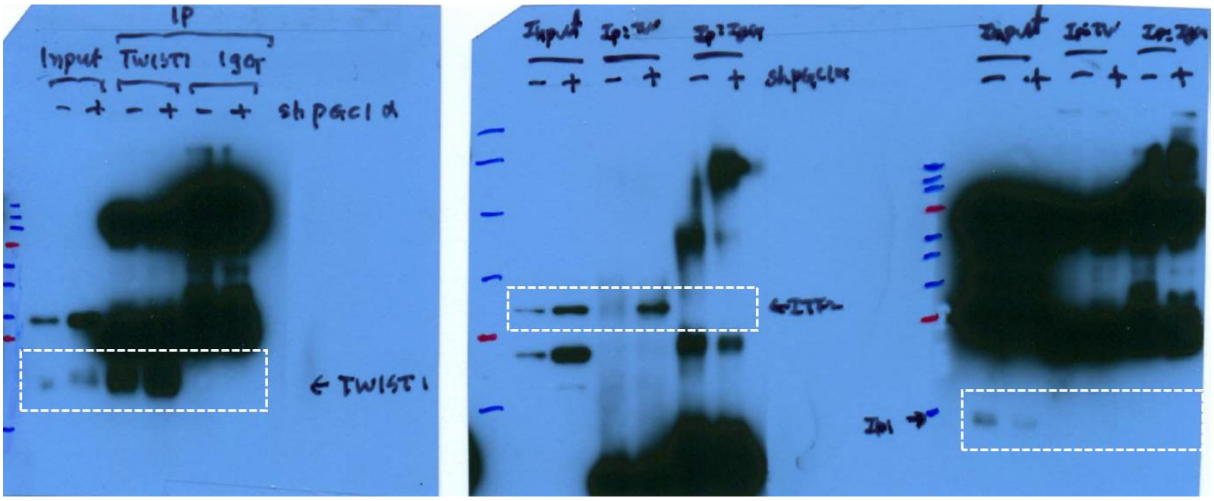

Figure 7G

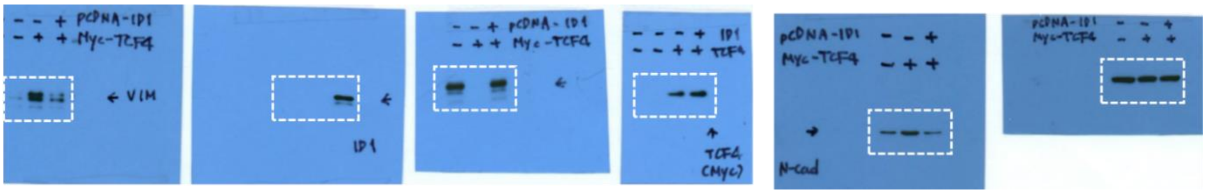

Figure S1

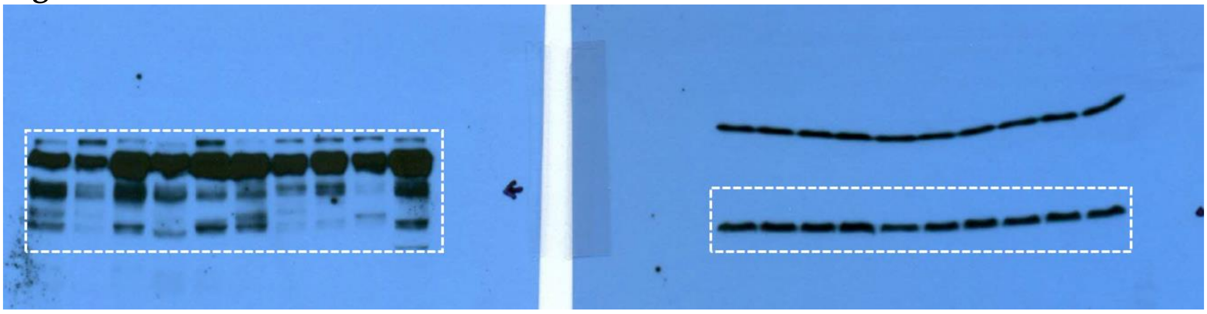

Figure S7. Whole western blot images.

High Resolution Images for Figure 2A

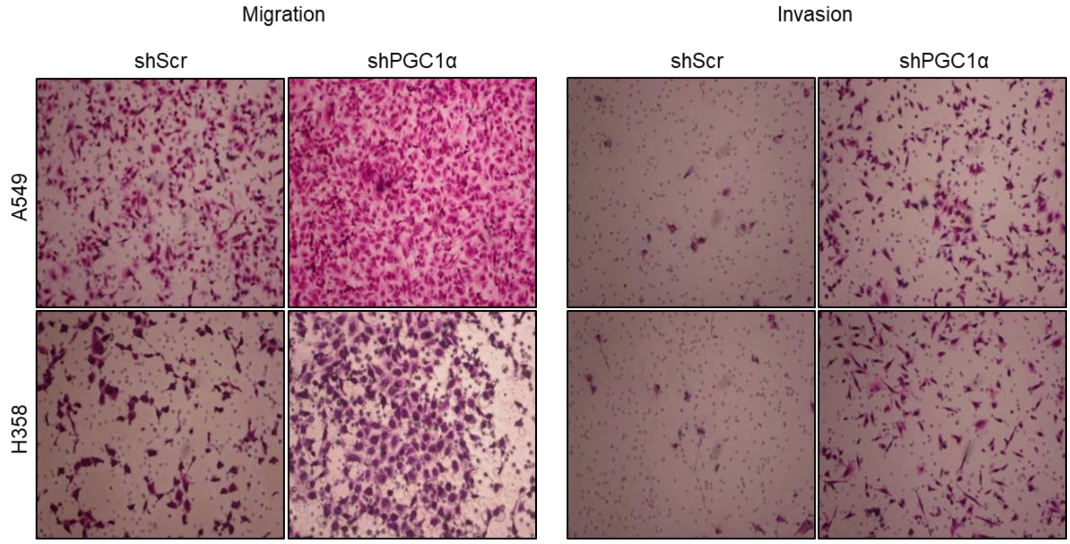

High Resolution Images for Figure 3C

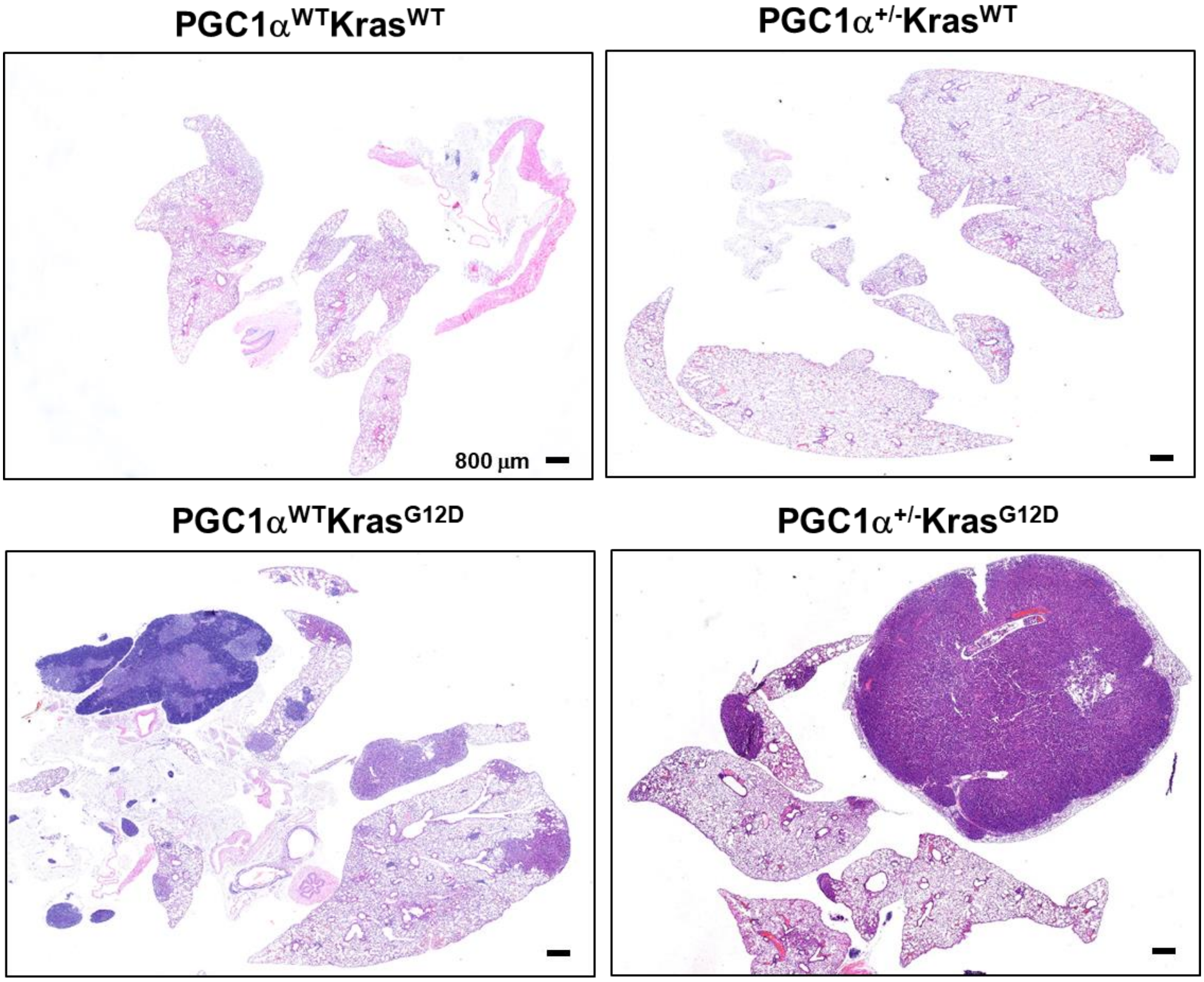

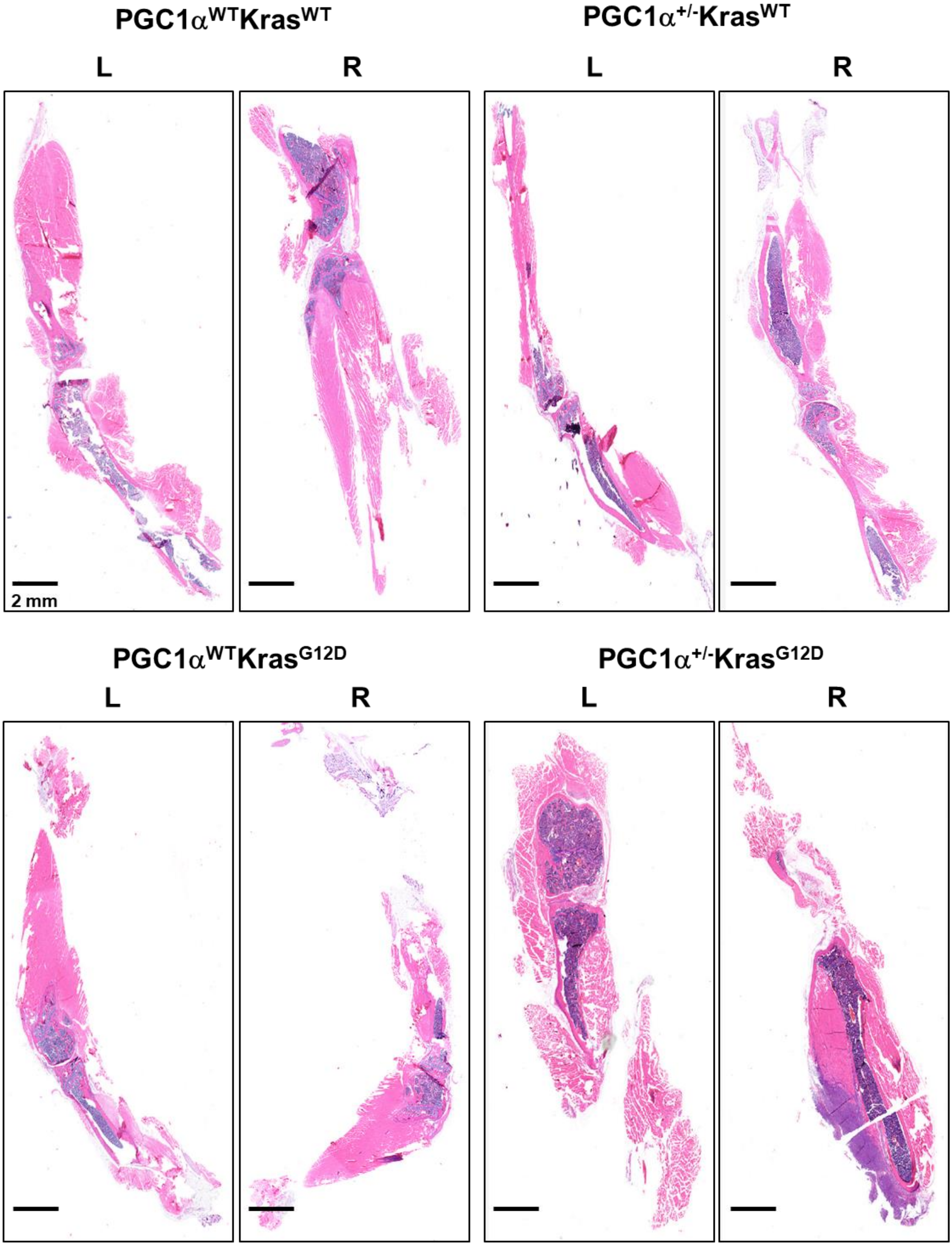
PGC1α^WT Kras^WT
L
R
PGC1α^+/- Kras^WT
L
R
2 mm
PGC1α^WT Kras^G12D
L
R
PGC1α^+/- Kras^G12D
L
R

High Resolution Images for Figure 5C

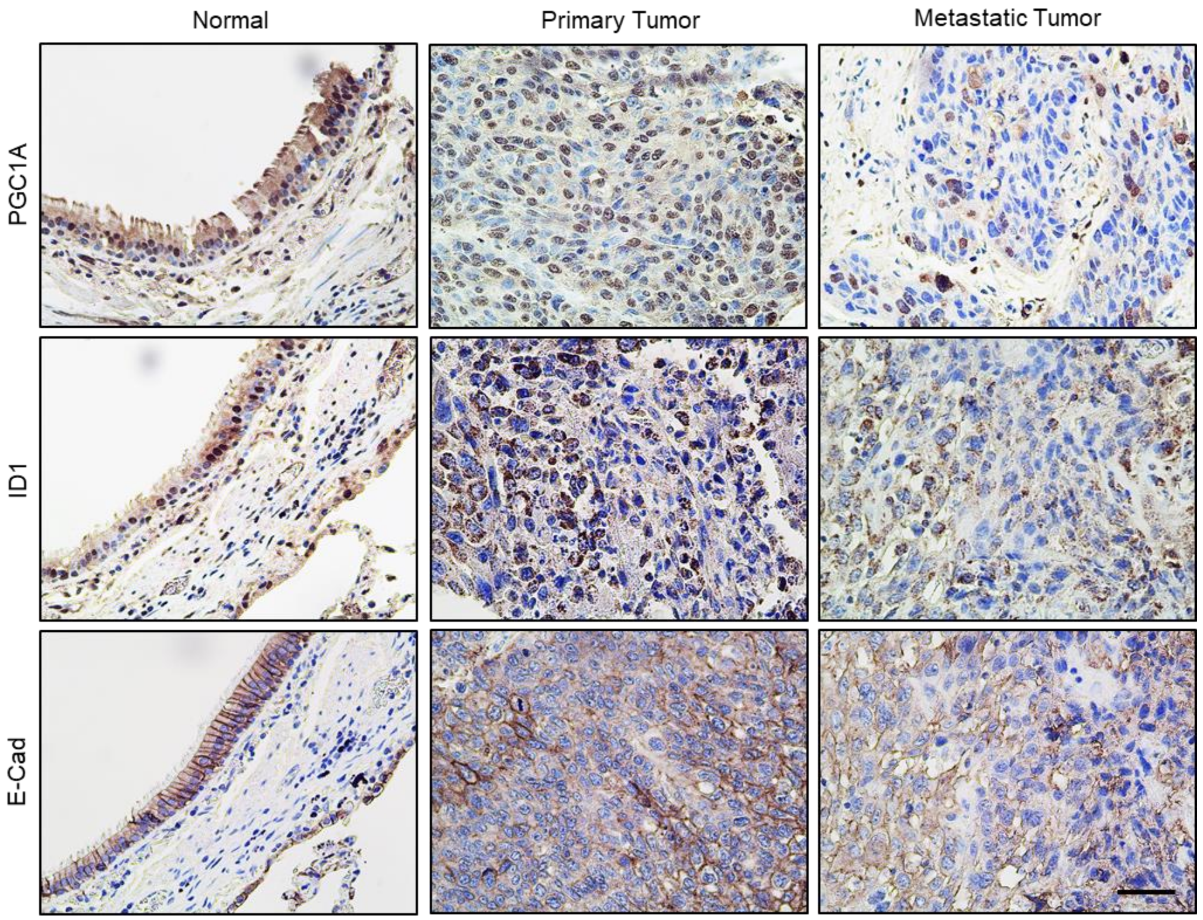

Figure S8. High Resolution Images of Figure 2A, Figure 3C and Figure 5C.

Table S1. Confirmation of lung cancer cell lines by using STR analysis.

Sample	D8S1179	D21S11	D7S820	CSF1PO	D3S1358	TH01	D13S317	D16S539
**A549 (control)**	13,14	29	8,11	10,12	16	8,9.3	11	11,12
**A549**	13,14	29	8,11	10,12	16	8,9.3	11,12	11,12
**NCI-H358 (control)**	13,14	28,30	10,11	11,12	14,18	6	8,12	12,13
**H358**	13,14	28,30	10,11	11,12	14,18	6	8,12	12,13
**Calu-1 (control)**	10	28	9,10	10	17	9,9.3	11,12	11
**Calu-1**	10	28	9,10	10	17	9,9.3	11,12	11

Sample	D2S1338	D19S433	Vwa	TPOX	D18S51	Amelogenin	D5S818	FGA
**A549 (control)**	24	13	14	8,11	14,17	X,Y	11	23
**A549**	24	13	14	8,11	14,17	X	11	23
**NCI-H358 (control)**	17,23	13,14	17	8,9	14	X,Y	10,12	20,21
**H358**	17,23	13,14	17	8,9	14	X,Y	10,12	20,21
**Calu-1 (control)**	16,17	13,15	15,16	8	14,17	X	10,12	20,21
**Calu-1**	16,17	13,15	15,16	8	14,17	X	10,12	20,21

Table S2. Clinical Information for Human Lung Cancer Patients.

No	Age	Sex	Organ	Diagnosis	Follow-up		Stage
					months	results	
1	63	M	Lung	squamous cell carcinoma	132	alive	Ⅰ B
2	63	M	Lung	squamous cell carcinoma	130	alive	Ⅰ B
3	50	M	Lung	squamous cell carcinoma	37	dead	Ⅱ A
4	67	M	Lung	squamous cell carcinoma	40	dead	Ⅱ A
5	54	M	Lung	squamous cell carcinoma	128	alive	Ⅰ B
6	39	M	Lung	squamous cell carcinoma	124	alive	ⅢA
7	71	M	Lung	squamous cell carcinoma	24	dead	Ⅱ A
8	67	M	Lung	squamous cell carcinoma	24	dead	Ⅱ A
9	65	M	Lung	squamous cell carcinoma	43	dead	ⅢA
10	47	M	Lung	adenocarcinoma	53	alive	Ⅱ A
11	38	M	Lung	adenocarcinoma	125	alive	Ⅱ A
12	48	F	Lung	adenocarcinoma	53	dead	Ⅰ A
13	67	M	Lung	adenocarcinoma	22	dead	Ⅰ B
14	55	M	Lung	bronchioloalveolar carcinoma	66	dead	Ⅰ A
15	69	F	Lung	bronchioloalveolar carcinoma	125	alive	Ⅰ B
16	52	F	Lung	bronchioloalveolar carcinoma	123	alive	Ⅰ B
17	34	M	Lung	bronchioloalveolar carcinoma	120	alive	Ⅰ B
18	47	M	Lung	adenosquamous carcinoma	131	alive	ⅢA
19	60	M	Lung	adenosquamous carcinoma	13	dead	ⅢA
20	60	F	Lung	small cell carcinoma	113	alive	Ⅱ A
21	65	M	Lung	small cell carcinoma	102	alive	Ⅰ B
22	60	M	Lung	combined small cell and adenocarcinoma	93	alive	Ⅱ B
23	43	M	Lung	large cell carcinoma	7	dead	ⅢA
24	56	M	Lung	large cell carcinoma	128	alive	Ⅱ A
25	63	M	Lung	large cell carcinoma	17	dead	Ⅱ A
26	37	M	Lung	mucoepidermoid carcinoma	131	alive	Ⅰ A
27	40	F	Lung	mucoepidermoid carcinoma	2	dead	Ⅳ
28	26	F	Lung	mucoepidermoid carcinoma	99	alive	Ⅰ B
29	45	M	Lung	squamous cell carcinoma	42	dead	ⅢB
30	59	M	Lung	squamous cell carcinoma	20	dead	ⅢB
31	62	M	Lung	squamous cell carcinoma	128	alive	ⅢA
32	39	M	Lung	squamous cell carcinoma	125	alive	Ⅱ A
33	58	M	Lung	adenocarcinoma	30	dead	Ⅰ B
34	54	M	Lung	adenocarcinoma	18	dead	Ⅱ A
35	62	F	Lung	adenocarcinoma	76	alive	ⅢB
36	72	M	Lung	mucinous adenocarcinoma	149	alive	ⅢA
37	67	F	Lung	adenosquamous cell carcinoma	29	dead	ⅢA
38	56	M	Lung	mucoepidermoid carcinoma	29	dead	Ⅱ A
39	45	M	Lymph node	metastatic carcinoma from #31	42	dead	IIA
40	59	M	Lymph node	metastatic carcinoma from #32	20	dead	IIIA
41	62	M	Lymph node	metastatic carcinoma from #33	128	alive	IB
42	39	M	Lymph node	matastatic carcinoma from #34	125	alive	IIB
43	66	M	Bone	metastatic carcinoma from #35	12	dead	IB
44	54	M	Lymph node	metastatic carcinoma from #36	18	dead	IIIA
45	62	F	Lymph node	metastatic carcinoma from #37	76	alive	IB
46	72	M	Lymph node	metastatic carcinoma from #38	149	alive	IB
47	76	F	Bone, femur	metastatic carcinoma from #39	12	dead	IIB
48	63	M	Soft tissue	metastatic carcinoma from #40	18	dead	IB
49	56	M	Lung	squamous cell carcinoma, well differentiated	46	dead	IIIA

50	34	F	Lung	adenocarcinoma, well differentiated	24	dead	IB
51	69	F	Lung	adenocarcinoma, well differentiated	118	alive	IIA
52	59	M	Lung	squamous cell carcinoma, moderately differentiated	117	alive	IIA
53	62	M	Lung	squamous cell carcinoma, moderately differentiated	22	dead	IB
54	42	M	Lung	bronchioloalveolar carcinoma, non-mucinous	16	dead	IB
55	64	M	Lung	adenocarcinoma, well differentiated	116	alive	IIA
56	64	M	Lung	squamous cell carcinoma, well differentiated	15	dead	IB
57	68	M	Lung	squamous cell carcinoma, moderately differentiated	6	dead	IIB
58	73	M	Lung	large cell neuroendocrine carcinoma	10	dead	IA
59	66	M	Lung	squamous cell carcinoma, well differentiated	7	dead	IIA
60	59	F	Lung	large cell carcinoma	4	dead	IB
61	59	M	Lung	squamous cell carcinoma, well differentiated	10	dead	IA
62	65	M	Lung	bronchioloalveolar carcinoma, non-mucinous	110	alive	IIIA
63	64	M	Lung	bronchioloalveolar carcinoma, mucinous	112	alive	IIB
64	61	M	Lung	squamous cell carcinoma, moderately differentiated	109	alive	IIA
65	69	F	Lung	squamous cell carcinoma, moderately differentiated	52	dead	IIA
66	64	M	Lung	squamous cell carcinoma, well differentiated	12	dead	IIA
67	74	M	Lung	squamous cell carcinoma, well differentiated	18	dead	IIA
68	53	F	Lung	squamous cell carcinoma, spindle cell	92	dead	IIIA
69	61	M	Lung	squamous cell carcinoma, well differentiated	46	dead	IIB
70	59	F	Lung	bronchioloalveolar carcinoma, mucinous	41	dead	IIA
71	58	M	Lung	large cell carcinoma	100	alive	IIIA
72	77	M	Lung	squamous cell carcinoma, moderately differentiated	20	dead	IIIA
73	53	F	Lung	bronchioloalveolar carcinoma, mucinous	76	dead	IB
74	72	M	Lung	squamous cell carcinoma, poorly differentiated	98	alive	IIA
75	63	M	Lung	squamous cell carcinoma, moderately differentiated	36	dead	IB
76	63	M	Lung	squamous cell carcinoma, moderately differentiated	97	dead	IIB
77	59	M	Lung	squamous cell carcinoma, moderately differentiated	97	alive	IB
78	62	M	Lung	squamous cell carcinoma, poorly differentiated	97	alive	IB
79	66	M	Lung	large cell neuroendocrine carcinoma	12	dead	IIA
80	58	M	Lung	squamous cell carcinoma, moderately differentiated	95	alive	IIIA
81	55	F	Lung	squamous cell carcinoma, poorly differentiated	79	dead	IIA
82	63	M	Lung	squamous cell carcinoma, well differentiated	3	dead	IB
83	81	M	Lung	squamous cell carcinoma, well differentiated	94	alive	IB
84	54	M	Lung	squamous cell carcinoma, moderately differentiated	25	dead	IIA
85	49	M	Lung	squamous cell carcinoma, well differentiated	91	alive	IIA
86	68	M	Lung	squamous cell carcinoma, poorly differentiated	21	dead	IIB
87	60	M	Lung	squamous cell carcinoma, poorly differentiated	90	alive	IIB
88	65	M	Lung	large cell carcinoma	90	alive	IB
89	51	M	Lung	bronchioloalveolar carcinoma, mucinous	90	alive	IIB
90	56	M	Lung	squamous cell carcinoma, well differentiated	25	dead	IB
91	46	M	Lung	squamous cell carcinoma, moderately differentiated	5	dead	IB
92	71	M	Lung	squamous cell carcinoma, moderately differentiated	89	alive	IIIA
93	69	M	Lung	squamous cell carcinoma, poorly differentiated	73	dead	IB
94	58	M	Lung	squamous cell carcinoma, well differentiated	88	alive	IIIA
95	62	M	Lung	bronchioloalveolar carcinoma, non-mucinous	41	dead	IIA
96	71	M	Lung	squamous cell carcinoma, well differentiated	10	dead	IIIA
97	65	M	Lung	squamous cell carcinoma, moderately differentiated	86	alive	IB
98	67	M	Lung	squamous cell carcinoma, poorly differentiated	108	dead	IIB
99	64	M	Lung	squamous cell carcinoma, poorly differentiated	6	dead	IB
100	69	M	Lung	squamous cell carcinoma, moderately differentiated	84	alive	IIIA
101	33	F	Lung	adenocarcinoma, moderately differentiated	83	alive	IB

102	60	F	Lung	adenocarcinoma, well differentiated	14	dead	IB
103	68	M	Lung	adenocarcinoma, moderately differentiated	80	alive	IIB
104	41	F	Lung	adenocarcinoma, moderately differentiated	20	dead	IB
105	67	M	Lung	normal lung (adjacent to cancer, match of #8)			
106	48	M	Lung	normal lung (adjacent to cancer, match of #12)			
107	34	M	Lung	normal lung (adjacent to cancer, match of #17)			
108	60	F	Lung	normal lung (adjacent to cancer, match of #20)			
109	59	M	Lung	normal lung (adjacent to cancer, match of #32)			
110	63	M	Lung	normal lung (apart from cancer, match of #1)			
111	55	M	Lung	normal lung (apart from cancer, match of #14)			
112	43	M	Lung	normal lung (apart from cancer, match of #23)			
113	58	M	Lung	normal lung (apart from cancer, match of #35)			

Table S3. Oligonucleotide sequences for short hairpin RNA, qRT-PCR, ChIP-PCR and gene cloning.

Oligonucleotides	Sequences (5′-3′)	IDENTIFIER
	**Oligomers for shRNA**	
shPGC1α #1	GCAGAGTATGACGATGGTATT	TRCN0000001165
shPGC1α #2	CCGTTATACCTGTGATGCTTT	TRCN0000001166
shID1 #1	CTACGACATGAACGGCTGTTA	TRCN0000274034
shID1 #2	CGGCTGTTACTCACGCCTCAA	TRCN0000019032
	**Primers for qRT-PCR**	
PGC1α Forward	CCTGTGATGCTTTTGCTGCTCTTG	
PGC1α Reverse	AAACTATCAAAATCCAGAGAGTCA	
CDH1 Forward	GACCGGTGCAATCTTCAAA	
CDH1 Reverse	TTGACGCCGAGAGCTACAC	
CDH2 Forward	CCACCTTAAAATCTGCAGGC	
CDH2 Reverse	GTGCATGAAGGACAGCCTCT	
VIM Forward	ATTCCACTTTGCGTTCAAGG	
VIM Reverse	CTTCAGAGAGAGGAAGCCGA	
ITGA5 Forward	AGGTAGACAGCACCACCCTG	
ITGA5 Reverse	CTCAGTGGAGTTTTACCGGC	
SNAI1 Forward	AGGTTGGAGCGGTCAGC	
SNAI1 Reverse	CCTTCTCTAGGCCCTGGCT	
SNAI2 Forward	TGACCTGTCTGCAAATGCTC	
SNAI2 Reverse	CAGACCCTGGTTGCTTCAA	
ID1 Forward	CTACGACATGAACGGCTGTTACTC	
ID1 Reverse	CTTGCTCACCTTGCGGTTCT	
ID2 Forward	GACAGCAAAGCACTGTGTGG	
ID2 Reverse	TCAGCACTTAAAAGATTCCGTG	
ID3 Forward	CTTCCGGCAGGAGAGGTT	
ID3 Reverse	AAAGGAGCTTTTGCCACTGA	
ID4 Forward	CCCTCCCTCTCTAGTGCTCC	
ID4 Reverse	GTGAACAAGCAGGGCGAC	
TCF4 Forward	CATAGGGAGTCCCATCTCCA	
TCF4 Reverse	GGACCAACTTCTTTGGCAAG	
PTHLH Forward	TTGTCATGGAGGAGCTGATG	
PTHLH Reverse	CGGTGTTCCTGCTGAGCTAC	
TGFBI Forward	GAAGGGAGACAATCGCTTTAGC	
TGFBI Reverse	TGTAGACTCCTTCCCGGTTGAG	
COL5A1 Forward	TCAGGCAAGTTGTGAAAATCT	
COL5A1 Reverse	CCATACCCGCTGGAAAGC	
NT5E Forward	TTGGAAATTTGGCCTCTTTG	
NT5E Reverse	ACTTCATGAACGCCCTGC	
MMP2 Forward	GGAAAGCCAGGATCCATTTT	

MMP2 Reverse	ATGCCGCCTTTAACTGGAG
H36B4 Forward	TGGTGATACCTAAAGCCTGGAA
H36B4 Reverse	CATGTTGCTGGCCAATAAGG
**Primers for ChIP-PCR**	
ID1 (-0.5kb) Forward	GCTTAGCTTCCTTGCCTCCT
ID1 (-0.5kb) Reverse	AGCCTCCTGCTCGTCTAGTG
ID1 (-4kb) Forward	TGAGACAGGGTCTTGCTTTG
ID1 (-4kb) Reverse	ATGAACCCAAGAAGTGGAGATT
CDH1 (-370~-277) Forward	CTCCAGCTTGGGTGAAAGAG
CDH1 (-370~-277) Reverse	GGGCTTTTACACTTGGCTGA
CDH1 (-169~+32) Forward	TAGAGGGTCACCGCGTCTAT
CDH1 (-169~+32) Reverse	TCACAGGTGCTTTGCAGTTC
CDH2 (+2999~+3112) Forward	AGGAGTGGAAGCAGAGCAGT
CDH2 (+2999~+3112) Reverse	GGCGTGTAAAGCAGACCATT
CDH2 (+11208~+11227) Forward	CTCCACTTCCACCTCCACAT
CDH2 (+11208~+11227) Reverse	GAGATCAAGGAGCTGGGGAG
**Primers for cloning**	
ID1-Luc -289 Forward	ATAGCTCGAGACCCTTGCTGTTCTGAAACC
ID1-Luc -289 Reverse	ACCGAAGCTTATGATTCTTGGCGACTGGCT
ID2-Luc -352 Forward	ATAGCTCGAGCCGCACTTACTGTACTGTAC
ID2-Luc -352 Reverse	ACCGAAGCTTCACACGCTGGAGCTTCCCTT

Table 4. Antibodies information for western blotting, chromatin immunoprecipitation (ChIP) and immunoprecipitation.

Antibodies	SOURCE	Cat No
anti-Histone H3 (K4-me3) rabbit polyclonal	Abcam	ab8580
anti-RNA polymerase II mouse monoclonal	Merck Millipore	05-623
anti-TCF4 mouse monoclonal	Abnova	H00006925-M04
anti-Twist1 mouse monoclonal	Abcam	ab50887
anti-PGC1α mouse monoclonal (4C1.3)	Merck Millipore	ST1202
anti-E-cadherin mouse monoclonal	BD Biosciences	610181
anti-N-cadherin mouse monoclonal	BD Biosciences	610921
anti-Vimentin (D21H3) rabbit monoclonal	Cell Signaling Technology	5741
anti-β-actin mouse monoclonal	Santa Cruz Biotechnology	sc-47778
anti-ID1 mouse monoclonal	Santa Cruz Biotechnology	sc-133104
anti-ID2 mouse monoclonal	Santa Cruz Biotechnology	sc-398104
anti-Flag mouse monoclonal	Sigma-Aldrich	F3165
anti-Myc-Tag rabbit monoclonal	Cell Signaling Technology	2278
anti-mouse IgG Peroxidase-goat	Jackson Immune Research	115-035-003
anti-Rabbit IgG Peroxidase- goat	Jackson Immune Research	111-035-003
Rabbit normal serum	Santa Cruz Biotechnology	sc-2027
Mouse normal serum	Santa Cruz Biotechnology	sc-2025
